# Supplementary material for: Paired DNA and RNA sequencing uncovers common and rare variation regulating human retinal gene expression
Source: Nat Commun. 2026 May 26;17:4595. doi: 10.1038/s41467-026-72979-4 (PMC13213049; doi:10.1038/s41467-026-72979-4)
Supplement: Supplementary file 1 — Supplementary Information [file 41467_2026_72979_MOESM1_ESM.pdf]

Paired DNA and RNA sequencing uncovers common and rare variation regulating human retinal gene expression

Supplementary Methods

1. **Gene expression quantification in Neurosensory Retina and Retinal Pigment Epithelium from RNA-Seq data**

1.1 Collection of Donor Eye Tissue

Donor eye tissues were obtained from the Manchester Eye Tissue Repository, an ethically approved Research Tissue Bank (UK NHS Health Research Authority, 15/NW/0932). Eye tissue was acquired after the corneas had been removed for transplantation and explicit written informed consent had been obtained from donors or their next of kin to use the remaining tissue for research. Guidelines established in the Human Tissue Act of 2004 (United Kingdom) and the tenets of the Declaration of Helsinki were adhered to. All ocular tissue was processed within 49 h postmortem. A sample from the iris and ciliary body was collected and stored at -80℃ and the lens and vitreous body were removed. The overlying neurosensory retina was checked for the presence of yellow foveal staining and a macular biopsy was extracted from one eye for further histological analysis. From the other globe, the entire neurosensory retina from the macula and peripheral regions were removed and immediately transferred to RNAlater, to stabilise and protect cellular RNA [1]. The underlying retinal pigment epithelium (RPE) was mixed with 250 µL phosphate buffered saline (PBS, Merck Millipore) and the cells were gently scraped from Bruch's membrane, working from the central macular region to the outer peripheral regions. Following centrifugation of the RPE-PBS mixture, the supernatant was removed and RNAlater was added to the RPE pellet and stored at -80℃.

1.2 RNA sequencing protocol

Total RNA was isolated using an Animal Tissue RNA Purification kit (#25700, Norgen Biotek), as per manufacturer's instructions. Sequencing was carried out by the Genomic Technologies Core Facility (GTCF) at The University of Manchester. Quality and integrity of the RNA samples were assessed using a 4200 TapeStation (Agilent Technologies) and sequencing libraries were generated using the *Illumina® Stranded mRNA Prep. Ligation* kit (Illumina, Inc.) according to the manufacturer's protocol. Briefly, total RNA (range of 200-500ng) was used as input material from which polyadenylated mRNA was purified using poly-T oligo-attached magnetic beads. Next, mRNA was fragmented under elevated temperature and then reverse transcribed into first strand cDNA. Following removal of the template RNA, second strand cDNA was then synthesized to yield blunt-ended, double-stranded cDNA fragments. Strand specificity was maintained by the incorporation of deoxyuridine triphosphate (dUTP) in place of dTTP to quench the second strand during subsequent amplification. Following a single adenine (A) base addition, adapters with a corresponding, complementary

thymine (T) overhang were ligated to the cDNA fragments. Pre-index anchors were then ligated to the ends of the double-stranded cDNA fragments to prepare them for dual indexing. A subsequent PCR amplification step was then used to add the index adapter sequences to create the final cDNA library which was amplified through 12 PCR cycles for each sample. The adapter indices enabled the multiplexing of the libraries, which were pooled in equimolar amounts to generate a final pool at a concentration of 10 nM prior to loading on an appropriate flow-cell.

. Paired-end sequencing (2x 75bp) was then performed on an Illumina NovaSeq6000 instrument. Finally, the output data was demultiplexed and BCL-to-Fastq conversion performed using Illumina's *bcl2fastq* software, version 2.20.0.422. To achieve desired depths of sequencing coverage, NSR samples underwent RNA Sequencing in 5 separate batches while RNA from RPE samples was sequenced in 3 separate batches.

1.3 RNAseq alignment and expression quantification

The Genotype-Tissue Expression (GTEx) analysis pipeline [2] was followed for quality control of the reads, alignment and expression quantification. All RNA-Seq data was processed uniformly. In summary, alignment of human reference genome GRCh38 was performed using STAR v2.7.4a [3]. The output BAM files were processed with Picard v2.27.1 to mark duplicate reads prior to gene expression quantification [4]. The GENCODE v38 annotation [5] was used for gene-level expression quantification. Each gene was collapsed to a single transcript using an isoform collapsing procedure designed by GTEX [2]. RNA-SeQC 1.1.9 [6] was used for gene-level expression quantification in read counts and transcripts per million (TPM). RSEM v1.3.0 [7] was used for transcript-level quantifications in transcripts per million. Read counts and TPM values were available for 60,000 genes in both NSR and RPE.

1.4 RNAseq quality control

Prior to sequencing, we assessed RNA concentration (ng / µl) and integrity (RNA Integrity Number – RIN) with the Agilent TapeStation system. Samples where RIN < 4 were excluded. After sequencing, we processed RNAseq BAM files (with marked duplicate reads) with RNASeQC to assess additional quality control metrics. These included the total number of reads, number of uniquely mapped reads, number of splice junctions, number of chimeric reads, read length and 3'/5' bias for all NSR and RPE samples. The 3'/5' bias metric was used to assess the integrity of the 5' end of quantified genes. It is defined as the ratio of sequencing depth between the 150 bp region at the 3' end of the gene and the 5' end of the gene (exclusively for genes over 600 bp with at least 5 unambiguous mapped reads). We excluded 5 RNA samples from our cohort (two NSR samples and three RPE samples) due to short read length (read length < 240 bp).

1.5 Cell type deconvolution of bulk RNAseq data

We used BayesPrism (Bayesian cell Proportion Reconstruction Inferred using Statistical Marginalization) [8] to run a deconvolution model to estimate the proportion of retinal cell types in our bulk RNA-seq data in NSR and RPE. We used single-cell RNAseq data from the ocular

posterior segment [9] as the reference dataset to train the model (study number: SCP2310, downloaded from https://singlecell.broadinstitute.org/single_cell/). The input single-cell dataset for BayesPrism was a cell-by-gene raw count matrix with cell type annotations generated for each cell and the bulk RNAseq dataset was a sample-by-gene raw count matrix. Genes expressed in fewer than 5 cells and those expressed at high magnitude were filtered from the scRNA-seq matrix using the built-in cleanup.genes function. The model was run on protein-coding genes only. The output of the deconvolution model was a set of posterior theta values for each sample in the bulkRNA dataset, corresponding to the estimated proportion of each cell type (from the scRNAseq matrix) that make up the sample.

1.6 Differential expression analysis between neurosensory retina (NSR) and retinal pigment epithelium (RPE)

1.6.1 Differential expression analysis using *deseq2* and *edgeR*

To ensure the validity of the transcriptomic datasets generated in this study, we assessed the biological relevance of expressed genes in NSR and RPE. We used the R package *deseq2* [10] to identify genes that were differentially expressed between NSR and RPE. We used the raw gene counts from RNASeQC as the input gene matrix. For this analysis, we excluded genes where mean TPM < 1 and/or had fewer than 10 reads in 20% samples across both tissues (n = 17,751 after filtering). Library normalisation was carried out using the default *deseq2* parameters. We included age and sex as covariates in the *deseq2* model. The false discovery rate threshold was set at 0.05. To assess the validity of *deseq2* results, we replicated the differential expression analysis using another R package, *edgeR* [11]. We excluded genes where mean TPM < 1 and utilised the default *edgeR* function *filterByExpr* to filter out genes with low expression by using an adaptive threshold based on library size (n = 17,718 after filtering). Library normalisation was carried out using a trimmed mean of M-values (TMM) algorithm. The false discovery rate was set at 0.05.

1.6.2 Gene Ontology Biological Process Gene Set Enrichment Analysis

To identify which gene ontology biological pathways were enriched in the upregulated genes in NSR/RPE, we carried out gene set enrichment analysis of the genes which were differentially expressed between both tissues according to the *deseq2* model (FDR < 0.05). This analysis was carried out using the *WebGestalt* R API [12]. The list of differentially expressed genes (Ensembl gene IDs) was used as input, alongside the log2 fold change values generated by *deseq2*. We applied Benjamini-Hochberg correction of p-values and applied an FDR threshold of 5%.

2. The output of WebGestalt was a set of Gene Ontology (GO) Biological Pathways which were enriched in each tissue. We used a clustering algorithm to group similar GO terms together and picked a representative term for each group. This analysis was performed with the R package *rrvgo* [13], which calculates the semantic similarity between a set of GO terms, and applies a clustering algorithm with a custom threshold. We used the

default threshold of 0.7. The representative term was set as the parent term with the greatest number of child terms. **Whole Genome Sequencing Data**

Whole genome sequencing of each donor was carried out in 7 separate batches. Five batches were sequenced at the Genomic Technologies Core Facility in Manchester and two batches were sequenced at Ocular Genomic Institute, Massachusetts Eye and Ear Infirmary, Harvard Medical School, Boston. All batches followed the same sequencing protocol and all WGS data was processed uniformly.

Sequencing libraries were generated using on-bead tagmentation chemistry with the Illumina® DNA Prep, (M) Tagmentation Kit (Illumina, Inc.) according to the manufacturer's protocol. Briefly, bead-linked transposomes were used to mediate the simultaneous fragmentation of gDNA (100-500ng) and the addition of Illumina sequencing primers. Next, reduced-cycle PCR amplification was used to amplify sequencing ready DNA fragments and to add the indices and adapters. Finally, sequencing-ready fragments were washed and pooled prior to paired-end sequencing (2 x 150bp) on an Illumina NovaSeq6000 instrument. Finally, the output data were demultiplexed and BCL-to-Fastq conversion performed using Illumina's bcl2fastq software, version 2.20.0.422. Genome alignment and variant calling was carried out using Illumina DRAGEN 4.0.3 with Machine Learning and Graph Map Enabled.

2.1 Aggregated VCF file and variant filtering

Aggregate variant calling was carried out using Illumina DRAGEN 4.0.3 Population Mode. Common variants were used for molQTL mapping (Minor Allele Frequency >2.5% and Minor Allele Count >10).

2.2 WGS sample-level quality control

We assessed WGS quality control statistics for each sample including median coverage, number of reads with Q>30, percentage genome >15x coverage, uniformity of coverage, total number variants, total number of SNVs, transition/transversion ratio and heterozygous/homozygous ratio. Outlier samples were excluded: one sample was excluded due to high number of variants and high het/hom ratio; and another sample was excluded due to high het/hom ratio.

2.3. WGS variant-level quality control

We applied quality control filters to the aggregate VCF to remove low-quality variant calls using a combination of *bcftools* (v.1.16) and *PLINK* (v.2.0). Firstly, variant calls which did not pass the DRAGEN hard filtering step were removed. Next, we filtered out variant sites which fell within low complexity regions defined by Krusche *et al.* [14]. Specifically, we removed variant sites which overlapped with all tandem repeats and homopolymers with 5bp slops added on each side (GRCh38_AllTandemRepeatsandHomopolymers_slop5.bed.gz). Subsequently, the remaining variant sites were decomposed and normalised using *bcftools norm* to convert all multiallelic variant sites to biallelic. We then removed variant calls where the inbreeding coefficient was lower than -0.3. Next, genotype calls with GQ<20 and/or where heterozygous

calls had allelic imbalance (AB >0.8 or AB <0.2) were set to missing. We also removed monomorphic and compound heterozygous variant calls (ALT1/ALT2), and variants that did not pass the Hardy-Weinberg Equilibrium test (p-value < 10^{-8}). Finally, we removed variant calls where missingness was greater than 20%.

2.4 WGS and RNAseq sample matching

To ensure concordance between paired WGS and RNAseq samples we excluded WGS-RNAseq pairs where the predicted relatedness was <0.8. Relatedness estimates were calculated using Somalier extract [15]. The default polymorphic sites recommended by Somalier were used to infer relatedness. We also assessed the proportion of singleton variants from each WGS sample which were also called from RNAseq data (read depth >15X) for each predicted WGS-RNA pair. Variants were called from RNAseq BAM files using *bcftools mpileup* and the depth at each variant site was calculated using *samtools depth.*

2.5 Genetic ancestry inference

We estimated the genetic ancestry of our cohort using the *somalier* ancestry inference feature [15]. We downloaded *somalier* genotype files from 2,504 samples from the 1000 Genomes Project phase 3 with corresponding super-population labels. *Somalier* calculated genotype principal components for samples from the 1000 Genomes Project and our cohort and estimated super-population ancestry groups based on a supervised machine learning algorithm built on a support vector machine (SVM).

3. Molecular phenotypes and covariates for cis-molQTL analysis

3.1 Formatting and normalisation of RNA-Seq data for expression-QTL analysis

For each tissue, genes which did not meet expression thresholds of >0.1 TPM in at least 20% of samples and ≥6 reads in at least 20% of samples were removed from eQTL analysis. Subsequently, expression values were normalised between samples using the trimmed mean of M-values normalisation (TMM) method [16] as implemented in edgeR [17]. For each gene, expression values were normalised across samples using an inverse normal transform as implemented by the GTEx analysis pipeline [2]. A normalised expression matrix was created for each tissue (NSR: 26,734 genes × 183 samples, RPE: 24,448 genes × 176 samples) and used as input for the eQTL analysis.

3.2 PEER Factors to capture hidden expression confounders.

To account for known and unknown biological and experimental confounding factors, a set of 30 covariates were generated for each RNA-Seq sample using the Probabilistic Estimation of Expression Residuals (PEER) method [18] applied to normalised gene expression levels. The PEER method is based on a Bayesian network which assumes that expression levels are influenced by additive effects from multiple independent sources [18].

3.3 Genotyping Principal Components

Principal component analysis with EIGENSOFT 6.0.1 (Patterson, Price and Reich, 2006) was carried out to capture ancestral variation within the cohort. The top five principal components for each participant were used as covariates in the eQTL analysis.

4. eQTL mapping with tensorqtl

TensorQTL [21] was used to find genetic variants which were significantly associated with the expression of nearby genes (up to 1 Mb away) in NSR and RPE. The required input files were the normalised gene expression matrix, the binary and filtered genotype data and a covariates table which included the following information for each participant: sex, WGS batch, five top principal components and 30 PEER factors. TensorQTL generates nominal p-values for each variant-gene pair based on a linear regression model between genotype and expression. Additionally, by using a permutation model, it can identify the variant which is most significantly associated with the expression of each gene.

4.1 Linear regression model for nominal gene-variant associations

For every input gene in NSR (n = 26,734 genes) and RPE (n = 24,448 genes), the following linear regression model was tested by TensorQTL for each variant within a 1 Mb-window (upstream and downstream) of its transcription start site:

Gene expression ~ Variant genotype + Sex + Five top Principal Components + 30 PEER Factors + WGS Sequencing Batch

Gene expression was assigned as the dependent variable and the variant genotype as the predictor of interest. However, gene expression quantification is dependent on multiple biological and technical factors. Therefore, additional covariates are included in the regression model. Nominal p-values were generated for each variant-gene pair by testing the alternative hypothesis that the effect size (or slope) of the variant genotype deviates from 0. To account for false positives, a false discovery rate (FDR) threshold was also calculated for each gene using a permutation scheme.

4.2 Permutation scheme to find candidate eQTL for each gene and False Discovery Rate threshold.

The aim of the permutation scheme was to find the best nominal association for each gene and assess its global significance [22]. TensorQTL implements a beta-approximation model to calculate the number of permutations for each gene [21]. This model is based on the hypothesis that P-values obtained through permutations are beta-distributed [23]. The beta-approximation model is based on estimating the shape parameters for the beta distribution of gene-variant p-values for each gene by maximum likelihood [24]. This estimation is calculated after generating a null set of P-values for each gene from a random number of permutations. The number of permutations required to generate the null set of P-values is lower than the number of permutations required to empirically calculate the beta distribution shape parameters, thus

reducing computational burden. Subsequently, the smallest nominal p-value can be assigned an adjusted beta-p-value.

Moreover, the adjusted beta p-values are used by TensorQTL to calculate q-values using the Storey and Tibshirani False-Discovery Rate (FDR) procedure (2003). Genes with a significant eQTL (eGenes) are those where FDR ≤0.05 for the most significant QTL association. To identify all significant variant-gene pairs for each eGene, a nominal p-value threshold ($\boldsymbol{p_t}$) was set based on the beta distribution model obtained from the permutations for that particular eGene [22]. Therefore, a significant association between an eGene and a nearby genetic variant (eVariant) is one where the nominal p-value $\leq p_t$, which corresponds to FDR ≤0.05. The genetic loci that are associated with the expression of a nearby eGene are referred to as cis-expression Quantitative Trait Loci (cis-eQTLs).

4.3 Allelic fold change

To quantify the eQTL effect size we estimated the log allelic fold change (aFC), following the method established by Mohammadi *et al.* [26]. The aFC is defined as the log-ratio between gene expression on the same chromosome as the eVariant ALT allele and the expression on the same chromosome as the REF allele.

4.4 Comparison with other eQTL studies

Intersection with GTEX eQTLs

All significant eQTL associations were downloaded from the GTEx Open Access portal (v8) for each available tissue (https://www.gtexportal.org/home/downloads/adult-gtex/qtl). We calculated the intersection between the number of METR-eQTLs which were also shared by each GTEx tissue. For an eQTL to be considered shared between both datasets, the variant ID (*CHR_POS_REF_ALT,* with GRCh38 coordinates) and the Ensembl gene ID (without version number) had to match. We compared the similarity between each GTEx tissue and the METR-eQTL dataset using the Intersection over Union (IoU) statistic. The IoU is the ratio of the number of eQTLs present in both sets over the total number of eQTLs in one set and/or the other. Therefore, the IoU statistic considers the different number of eQTLs present in each GTEx tissue. We also calculated the number of shared eGenes between each GTEx tissue and the METR-eQTL dataset, as well as the degree of eGene similarity using the IoU statistic.

Intersection with EyeGEx,the Eye eQTL Atlas and Strunz *et al.*

We compared all METR eQTLs with retina eQTLs mapped by EyeGEx [27] and Strunz et al. [28]. METR variant IDs were converted to rsIDs using dbSNP build release 156 [29]. First, we identified genes had been associated with eQTLs in our study and in EyeGEx and/or Strunz *et al.* [28] (common eGenes). For these genes, we extracted the top eQTLs identified by EyeGEx and/or Strunz *et al.* [28] and checked if they were replicated in our cohort or if they were in high LD (r2 > 0.8) with a METR-NSR eQTL. Pairwise LD scores were calculated using LDlinkR [30].

Additionally, we queried the METR-eQTL dataset to understand if we replicated eQTLs that had been identified through TWAS and colocalization analyses as candidate variants which

impact AMD risk genes [27,31]. We also tested if the candidate variants were in high LD with METR-eQTLs or if novel METR-eQTLs had been identified for the risk gene. We downloaded eQTLs from [31] from the eye eqtl browser, available at (https://eye-eqtl.com/).

5. Annotation of eVariants and bootstrapping analysis to calculate enrichment of eQTLs in characterised regulatory loci

All NSR and RPE eQTL variants were annotated with the Ensembl Variant Effect Predictor [32]. We also assessed overlap and annotated all eVariants with a set of tissue-specific and cell-type specific annotations of candidate cis-regulatory elements (cCREs) from a variety of sources (*Supplementary Table 1*). These included characterised regulatory loci from retina, RPE and macula from Cherry *et al.* [33], derived from bulk ATAC-seq and H3K27ac ChIPseq. We also included cell-type specific regions of open chromatin detected by scATACseq from retina samples published by [34]. We annotated eVariants with non-eye specific cCREs from adult tissues in EpiMap [35]. Specifically, we downloaded all enhancer and promoter BED files from all adult tissue types from https://compbio.mit.edu/epimap/. We excluded cCREs from embryonic and neonatal cell types, and all primary cell lines as their regulatory patterns may be different from adult human tissues. Finally, we used cell-type agnostic candidate cis-regulatory element (cCRE) annotations from the ENCODE Encyclopaedia of cCREs [36], which included promoters, proximal/distal enhancers, CTCF-only binding sites and DNase-H3K4me3 sites (poised elements).

To calculate the relative enrichment of eVariants which overlapped with each type of regulatory element, we used bootstrapping analysis. We carried out 1,000 subsampling iterations. For each iteration, 100,000 eVariants were randomly selected with replacement. We then matched each eVariant with another non-eQTL variant from our cohort that had a similar allele frequency and gene density (number of gene TSSs within 1Mb of the variant). Each subset of eVariants and matched control variants was intersected with the cCRE datasets described above using BEDtools [37]. We then calculated the relative enrichment as the ratio of eVariants to control variants which overlapped each type of cCRE. For each cCRE type we then calculated the mean relative enrichment and the 95% confidence interval. If there was no relative enrichment, we would expect the mean score to equal 1. Therefore, to calculate significance p-values from the bootstrapped results we created a null distribution where *mean = 1* and *std. deviation = std. deviation of the bootstrapped result set*. We then calculated the Z-score between the observed bootstrapped mean and the mean of the null distribution.

6. Analysis of the properties of eQTLs that impact known eye disease-related genes

To understand if there were trends that were specific to eQTLs associated with known monogenic eye disease genes, we utilised the EyeG2P resource [38]. We identified which METR-eGenes were described in EyeG2P as causes of rare monogenic disorders and

classified these as eye-disease genes. All other METR-eGenes were considered non-eye disease genes. Subsequently we compared the following eQTL/eGene properties between eye-disease and non-eye disease gene eQTLs:

- Gene expression variability, measured by the coefficient of variation (CoV). The CoV was calculated by dividing the standard deviation over mean gene expression (TPM).
- Number of eQTLs associated with each eGene.
- The eQTL effect size, measured by the absolute log2 allelic fold change (see section 4.3).
- The allele frequency of the eQTL variant from gnomad v4

Additionally, to account for the fact that genes associated with rare monogenic eye disease had higher mean expression than non-eye disease genes, we adopted a bootstrapping approach to compare eQTL properties, while controlling for mean gene expression. We carried out 1000 bootstrapping iterations. In each iteration, the following steps were carried out:

1. Randomly selected 100 eQTLs associated with eye disease genes.
2. Randomly selected 100 eQTLs associated with non-eye disease genes, but the mean gene expression (+-5%) of the associated eGene was matched to the mean expression of the eye disease genes associated with the 100 randomly selected eQTLs in step 1.
3. Calculated the median expression and coefficient of variation for the eGenes associated with the eQTLs in subsets 1) and 2), and calculated the median gnomad AF and log2 AFC for the eQTL variants selected in subsets 1) and 2).
4. Appended the medians to a master array for the eye disease and non-eye disease eQTLs.

Therefore, the final product after running the 1000 iterations was two sets of arrays containing the median values for the eGene expression, eGene coefficient of variation, eQTL AF and eQTL effect size for disease gene and non-disease gene eQTLs. We then calculated the median and the 95% confidence intervals of the bootstrapped arrays.

7. Identification of transcriptome outliers using the DROP pipeline

We utilised the DROP v.1.4.0 snakemake pipeline to identify transcriptome outliers from NSR and RPE. We ran the aberrant expression module with the parameters listed in *Supplementary Table 2* to identify expression outliers. The aberrant splicing and monoallelic expression modules were applied to NSR samples with the parameters listed in *Supplementary Table 3* and *Supplementary Table 4* respectively.

8. Hierarchical workflow to identify candidate variants driving outlier expression

We designed a hierarchical workflow to identify candidate variants driving outlier expression (eOutliers) using snakemake version 7.32 (*Supplementary Figure 14)*. Each eOutlier was identified by a unique sampleID-geneID combination. Briefly, the workflow would first identify a pLoF variant from the eOutlier sample in the corresponding eOutlier gene, which could be an exonic structural variant, or a SNV with a high impact consequence based on *Ensembl's Variant Effect Predictor (v.112.0).* High impact SNVs included nonsense, frameshift, start/stop loss and canonical splice donor/acceptor variants. If no pLoF variant could be identified, the workflow would then search for regulatory variants which were within 10Kb of the eOutlier gene body. Regulatory variants were defined as structural variants and rare SNVs which overlapped with nearby retina cCREs or non-retina specific cCREs from different adult tissues in EpiMap. If no regulatory variant was identified, the model would check if any other non-coding structural variant fell within 10Kb of the eOutlier gene body, before returning a negative search result. All intersection steps were carried out using *bedtools intersect.* We used *bcftools* to extract SNVs from each outlier sample for the corresponding eOutlier gene from the filtered aggregate VCF. Rare SNVs were defined as those where the gnomad v4 allele frequency was lower than 1%.

The output of the workflow is a JSON object, where each eOutlier ID is assigned a category based on the candidate variant type (*Supplementary Table 5)*, alongside details of the eOutlier metrics from OUTRIDER and a list of the candidate variant/s for further analysis.

For all genes with an eOutlier in the NSR (n = 702), we extracted all rare variants (gnomAD allele frequency < 1%) which intersected with the gene body ± 10Kb. Variants were extracted for all samples with NSR RNAseq data (n = 183) from the post-QC aggregate VCF. We then annotated all rare variants with selected annotations from VEP [32] and CADD [39] *(Supplementary Data 6)* and intersected them with known retina-specific cCREs from Cherry et al. [33] and non-retina specific cCREs from EpiMap. Missing annotations were replaced with default imputation values obtained from the CADD release notes (*Supplementary Data 6)*.

The scripts required to run the Watershed model were downloaded from https://github.com/BennyStrobes/Watershed. Each line of the input dataset corresponded to a single gene-sample pair and included a set of annotations describing the rare variants nearby the gene and the p-values corresponding to the aberrant expression, aberrant splicing and monoallelic expression modules from DROP for NSR and RPE. From this data, the model was able to optimise the appropriate parameters using Watershed Exact optimisation and then calculate the posterior probabilities that a particular set of genomic annotations would lead to each significant eOutlier p-value (adjusted p-value < 0.05).

For each gene-sample, Watershed required a single value for each annotation listed on *Supplementary Data 6*, even though more than one rare variant may lie within the gene of interest. Therefore, from the set of annotated rare variants corresponding to a particular gene-sample, we selected the annotation which was considered the most informative (generally the maximum value). Therefore, each line of the input dataset for Watershed described the landscape of rare variants which surrounded a particular gene. Watershed also required N2 pairs, or pairs of individuals which shared the same rare variant to evaluate the model. We randomly selected 3000 N2 pairs from our dataset.

The Watershed model was run using the predict_watershed.R script with an adjusted p-val threshold of 0.05 and the number of dimensions set to 6 (corresponding to the 3 DROP p-values from NSR and 3 from RPE).

9. Dual reporter luciferase assay

10.1 Plasmid construction for *CAND2* luciferase reporter assay

A 294bp fragment of the wild-type promoter region from *CAND2* was PCR-amplified from control genomic DNA using Phusion High-Fidelity DNA Polymerase (Promega). To introduce variants, two overlapping fragments were amplified using combination of mutagenic primers. Variants constructed were the variant of interest, NM_001162499.2:c.-41A>G, and a variant that is common in the general population and not expected to impact *CAND2* expression, NM_001162499.2:c.-36C>T. The primers listed in ***Supplementary Table 6*** were used.

The wild-type and variant fragments were assembled into *Nhe*I-*Nco*I digested pGL4.10[luc2] firefly luciferase plasmid using the Gibson method. The assemblies were transformed into competent *E. coli* grown overnight on LB agar containing carbenicillin. Candidate colonies were picked for culture and plasmid isolation. The plasmid constructs were verified by Sanger sequencing.

10.2 Transfection and Luciferase Reporter Assay

Human K562 cells were maintained under standard tissue culture conditions in RPMI-1640 medium (Sigma-Aldrich) supplemented with 10% Fetal Bovine Serum (Sigma-Aldrich) and incubated at 37°C and with 5% CO_2. For the luciferase assay, the cells were grown in 500 µL medium in tissue-culture treated 24-well plates at 37°C and with 5% CO_2. Cells were transiently transfected with 500 ng of plasmid using Lipofectamine LTX (Invitrogen) following the manufacturer's standard protocol. Empty pGL4.10[luc2] plasmid was transfected as a control for background activity. The *Renilla* luciferase pGL4.74[hRluc/TK] vector (Promega) was co-transfected as an internal luminescence control.

Following 20-24 hr incubation at 37°C with 5% CO_2, a dual luciferase assay was conducted using the Dual-Glo® Luciferase Assay (Promega). The relative luciferase units (RLU) were calculated by dividing the firefly luciferase value by the *Renilla* luciferase value after subtracting the background luciferase value. A relative response ratio (RRR) was calculated using the wild-type as the reference. Each assay was performed with three technical replicates and performed on 3 separate occasions.

One way ANOVA statistical analysis with multiple comparison test (Dunnett’s) was performed using GraphPad Prism, to understand the impact of variants on luciferase expression.

Supplementary Tables

Supplementary Table 1 Summary of annotations of non-coding regions used in the enrichment analysis

Dataset	Description
Cherry et al. [33]	cCREs in retina and RPE calculated by intersecting bulk ATACseq with H3K27ac ChIPseq.
Wang et al. [34]	Regions of accessible chromatin from Retina scATACseq, comprising 8 different retina cell types
EpiMap [35]	Tissue specific cCREs from 18 different adult tissue types.
ENCODE [36]	Cell type agnostic regulatory elements, classified into promoters, proximal/distal enhancers, CTCF-only and poised cCREs

Supplementary Table 2 Parameters used to run the aberrant expression module on DROP 1.4.0

Parameter	Value
Groups	NSR, RPE
FPKM Cutoff	1
Implementation	Autoencoder
Z-score cutoff	0
p-adj cutoff	0.05
Max tested dimension proportion	3
Yield size	2,000,000

Supplementary Table 3 Parameters used to run the aberrant splicing module on DROP 1.4.0

Parameter	Value
Groups	NSR
recount	FALSE
longRead	FALSE
keepNonStandardChrs	FALSE
filter	TRUE
minExpressionInOneSample	20
quantileMinExpression	10
minDeltaPsi	0.05
FRASER_version	FRASER2
deltaPsiCutoff	0.1
quantileForFiltering	0.75
Implementation	PCA
p-adj cutoff	0.05
Max tested dimension proportion	6

Supplementary Table 4 Parameters used to run the monoallelic expression module on DROP v.1.4.0

Parameter	Value
Groups	NSR
gatkIgnoreHeaderCheck	TRUE
padjCutoff	0.05
allelicRatioCutoff	0.8
addAF	TRUE
maxAF	0.001
maxVarFreqCohort	0.05

Supplementary Table 5 Output categories for each eOutlier based on the identied candidate variants driving outlier expression

eOutlier category based on the candidate variant	Variant type
SV_CNV_intersect_whole_gene	pLoF variant
SV_CNV_intersect_exon	pLoF variant
High_impact_SNV_disrupt_gene	pLoF variant
SV_CNV_intersect_Eye_cCRE	Regulatory variant
Rare_SNV_Intersect_Eye_cCRE	Regulatory variant
SV_CNV_intersect_EpiMap_cCRE	Regulatory variant
Rare_SNV_Intersect_EpiMap_cCRE	Regulatory variant
SV_CNV_intersect_intron	Non-coding structural variant
SV_CNV_within_10Kb_gene_body	Non-coding structural variant
Common_SNV_Intersect_cCRE	No candidate variant
No_SV_or_SNV_of_interest	No candidate variant

Supplementary Table 6 Primers used for the plasmid construction for the CAND2 luciferase reporter assay

Primer name	Primer sequence (5’-3’)
**Frag1-for**	CTGGCCGGTACCTGAGCTCGGTCTCTGGGGGTGTGTGCA
**Frag1 c.-41A>G -rev**	GAGGGAATACGGCGGCGCGGGCGCCCCCTCCCTCG
**Frag2 c.-41A>G -for**	GGGCGCCCGCGCCGCCGTATTCCCTCCCG
**Frag1 c.-36C>T -rev**	CGGGAGGAAATATGGCGGCGCGGGCGCCC
**Frag2 c.-36C>T -for**	GGGCGCCCGCGCCGCCATATTTCCTCCCG
**Frag2-rev**	TCTTAATGTTTTTGGCATCTTCCATGGTGGCTGCGC

Supplementary Figures

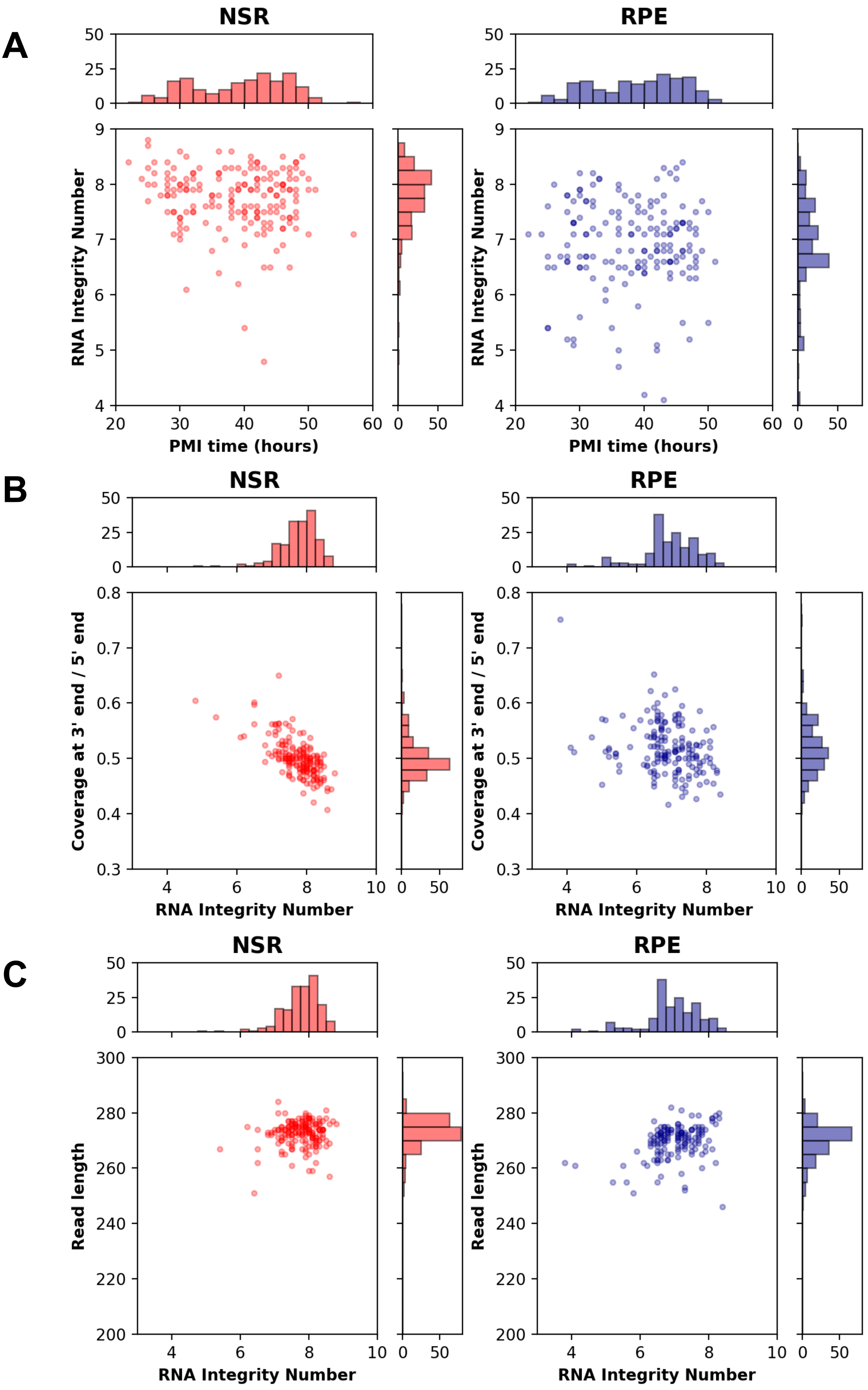

Supplementary Figure 1 Quality control metrics for NSR (N=183) and RPE (N=176) RNASeq samples. *A) The relationship between PMI time and RNA integrity numbers. We did not observe a noticeable decrease in RNA integrity in samples with a high PMI time (>40 hours). B) The relationship between RNA Integrity Numbers and 3'/5' bias in RNASeq samples C) The relationship between RNA Integrity Numbers and RNASeq read length in bp*

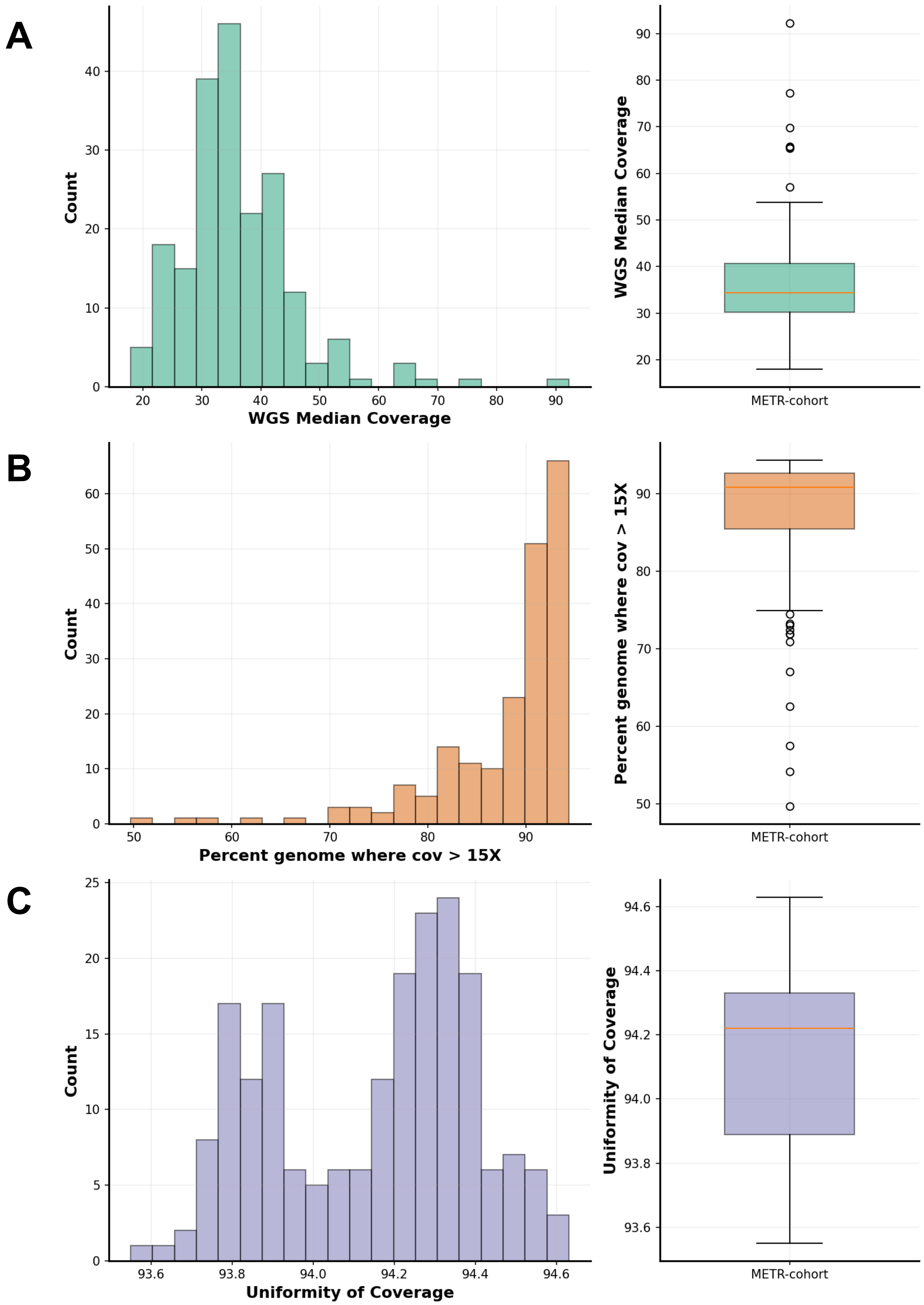

Supplementary Figure 2 Whole genome sequencing quality control metrics (n=201) *A) Median genome-wide coverage values B) Percentage of the genome covered by at least 15 reads C) Uniformity of Coverage. Box plots show the median (centre line), interquartile range (box; Q1–Q3), and whiskers extending to the most extreme data points within 1.5×IQR. Points beyond the whiskers are plotted as outliers.*

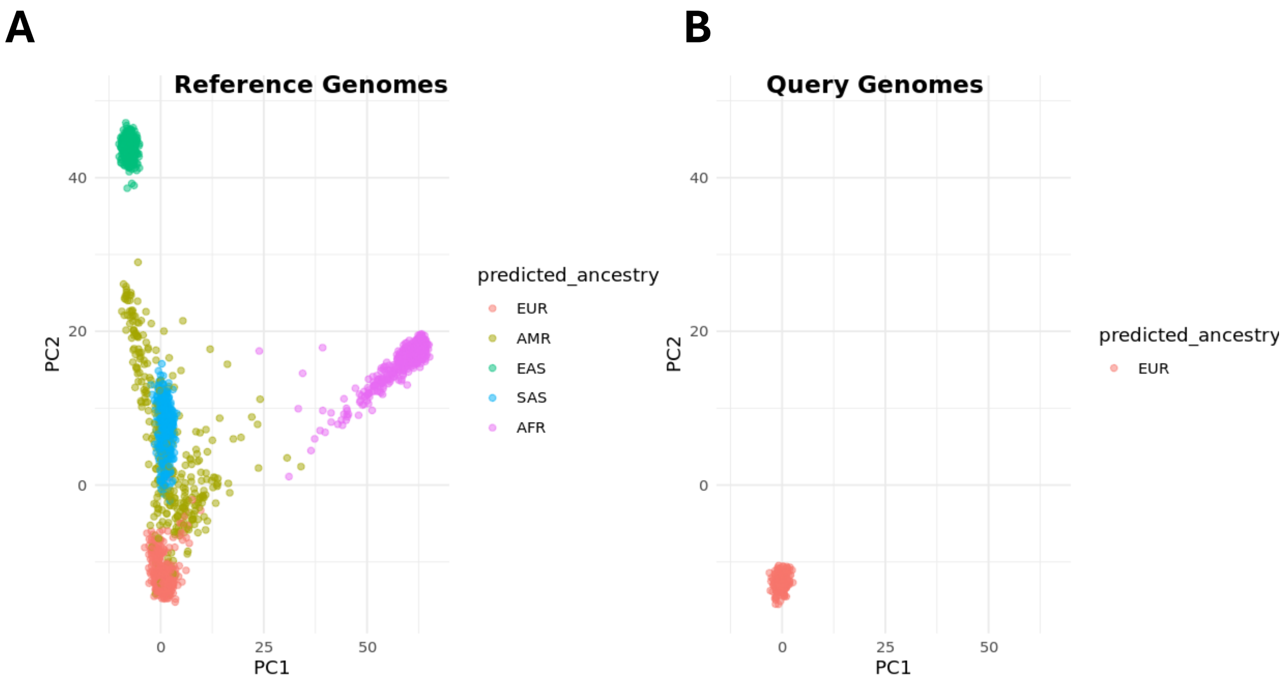

Supplementary Figure 3 Inferred genetic ancestry of the METR-cohort generated by somalier A) *Genotype principal components for participants of the 1000 Genomes Project phase 3 with corresponding super-population labels.* ***B)*** *Genotype principal components for participants of the METR cohort (n=201) which were projected alongside the 1000 Genomes Project participants, with the inferred genetic ancestry label generated by somalier.*

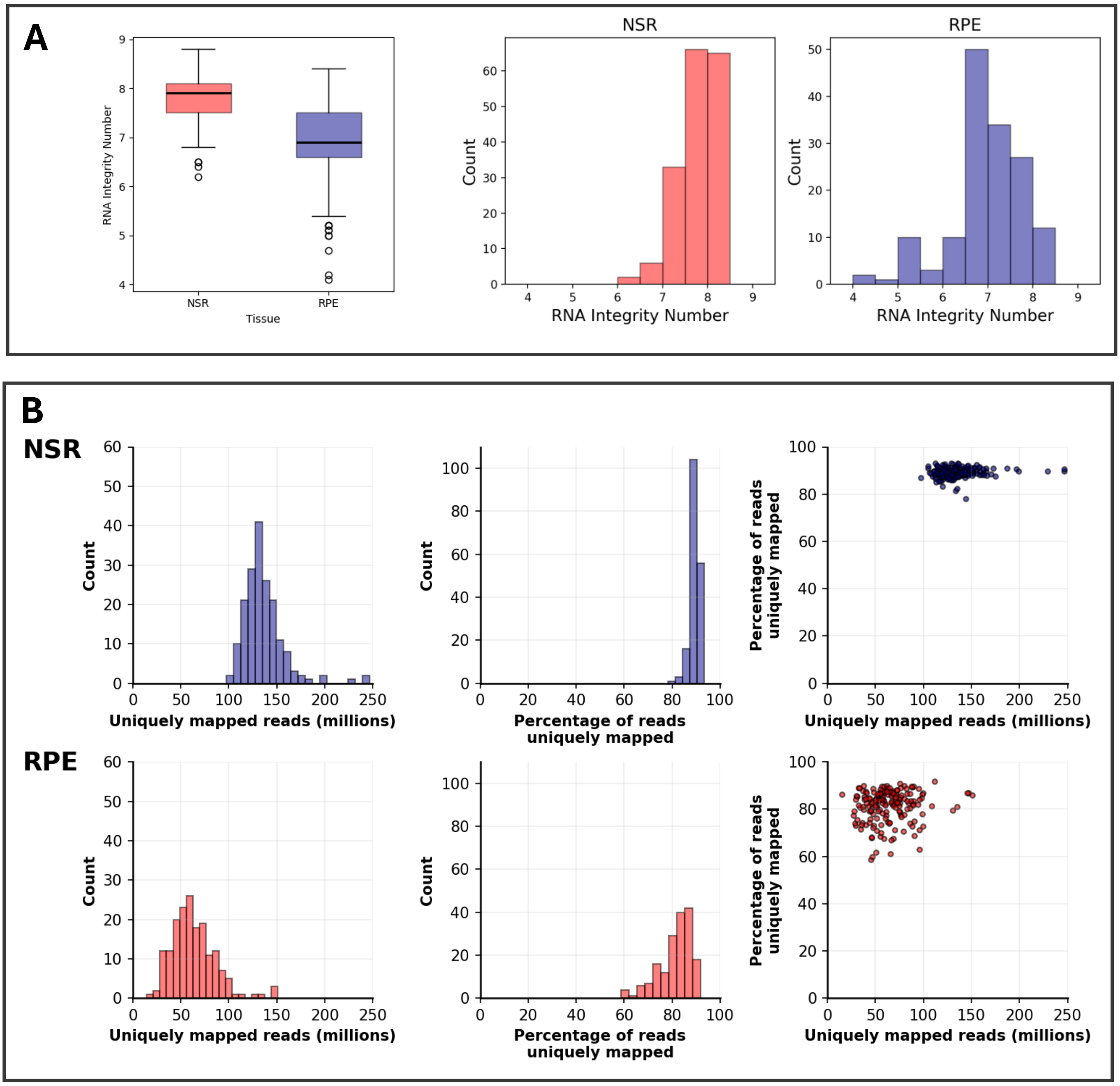

Supplementary Figure 4 *A) Distribution of RIN scores in NSR (n = 183) and RPE (n = 176) RNASeq samples. Box plots show the median (centre line), interquartile range (box; Q1–Q3), and whiskers extending to the most extreme data points within 1.5×IQR. Points beyond the whiskers are plotted as outliers. B) Number of uniquely mapped reads in NSR (n = 183) and RPE (n = 176) samples from the METR-GT cohort and the mapping rate (percentage of reads uniquely mapped)*

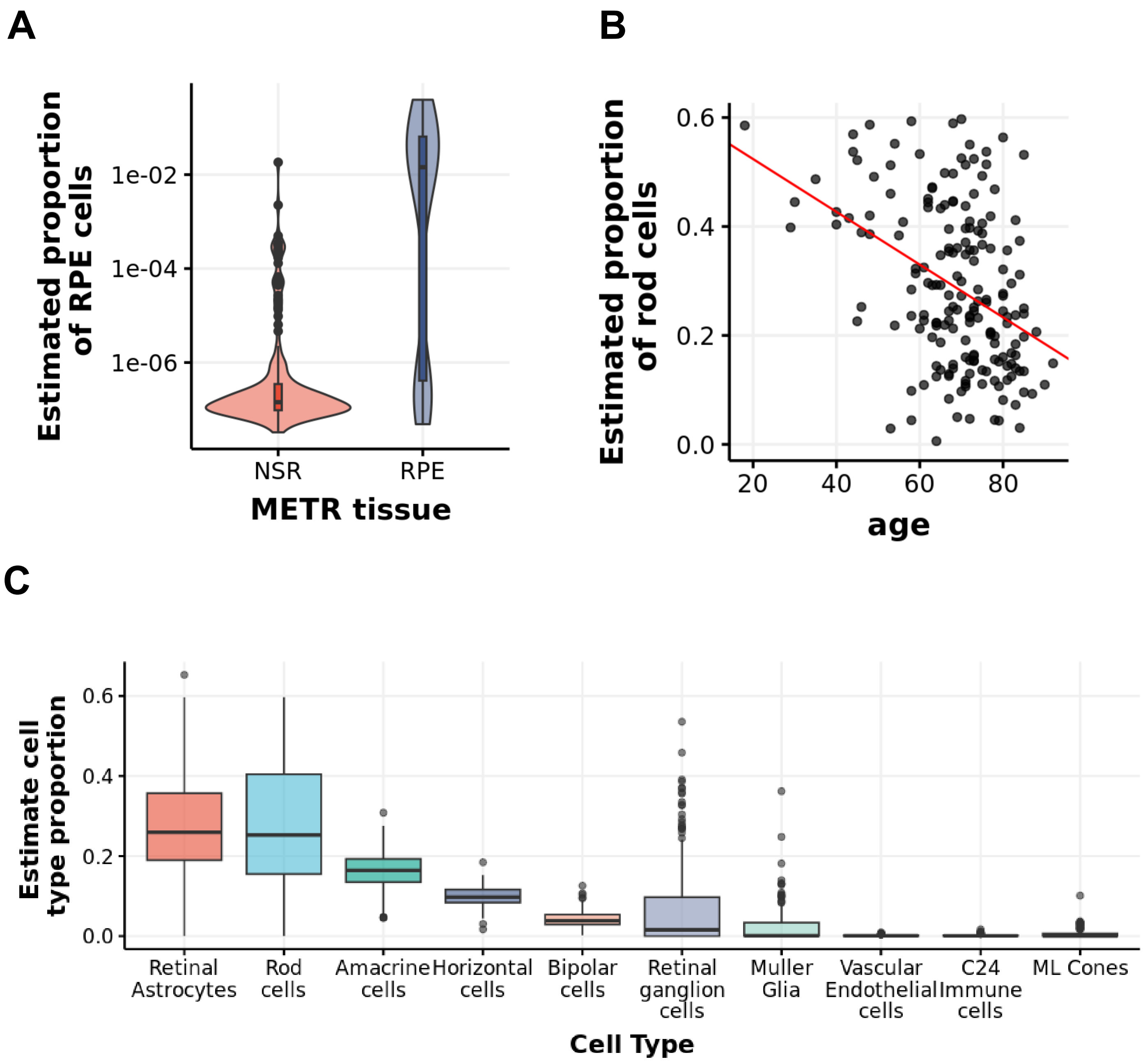

Supplementary Figure 5 Cell type deconvolution of METR bulk RNASeq samples *A. The estimated proportion of RPE cells in METR-RPE samples (n = 176) is higher than in the METR-NSR (n = 183) cohort. B. The estimated proportion of rod cells decreases in samples from older donors. C. The estimated proportion of individual cell types across the METR-NSR cohort. Box plots show the median (centre line), interquartile range (box; Q1–Q3), and whiskers extending to the most extreme data points within 1.5×IQR. Points beyond the whiskers are plotted as outliers.*

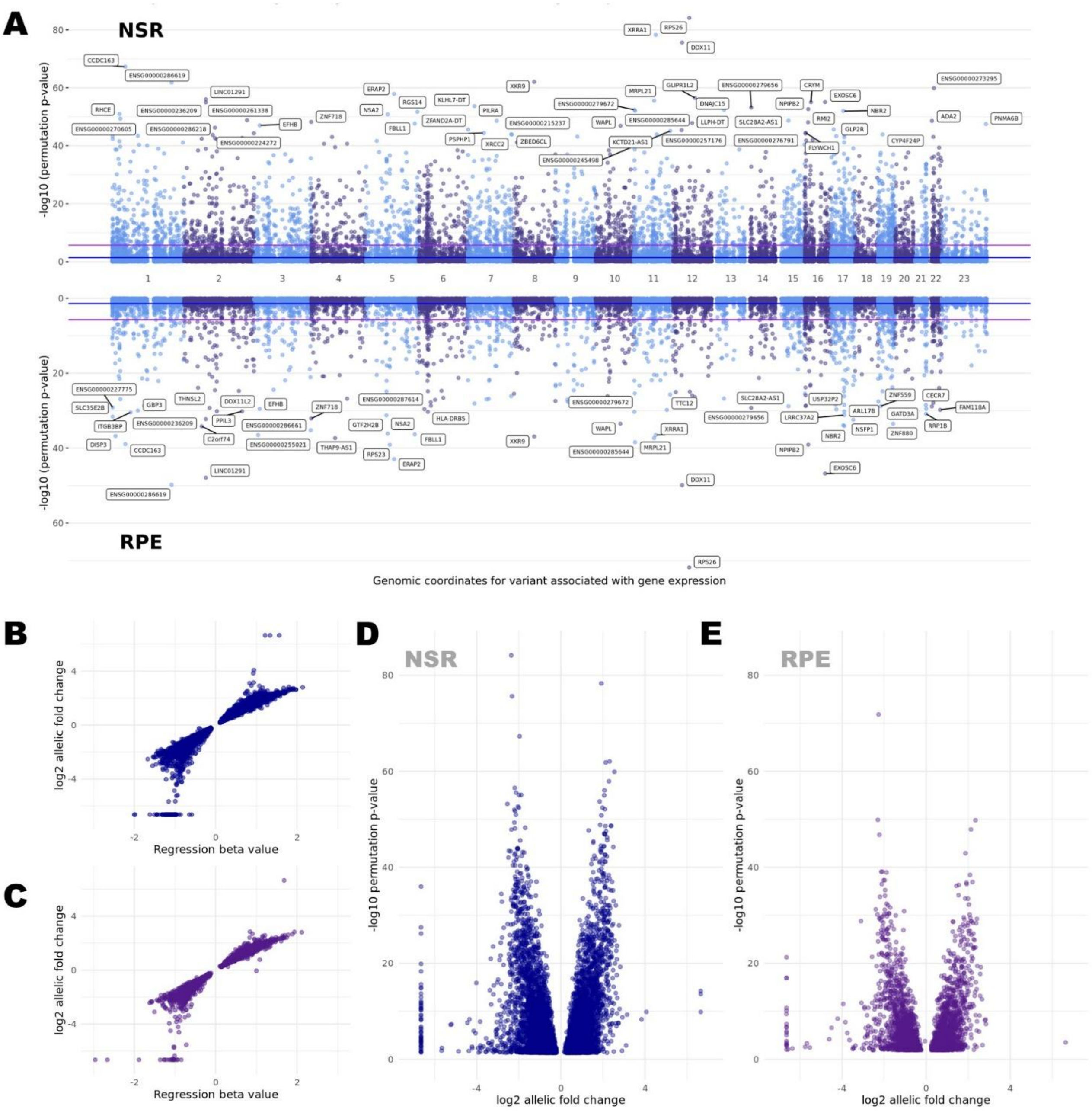

Supplementary Figure 6 Cis-eQTL mapping in NSR (n = 183) and RPE (n=176) based on a permutation model to identify the top eQTL candidate per gene. A) *Manhattan plot of the top cis-eQTLs per gene in the NSR (top) and the RPE (bottom). The blue line indicates the False Discovery Rate (FDR) threshold of 5% used to identify genes with a significant eQTL (eGenes). In the NSR we identified 8,609 eGenes and 3,229 in the RPE. The purple line indicates the more conservative Bonferroni corrected p-value threshold 0.05).* ***B)*** *Relationship between the regression beta values computed for each of the top eQTLs in the NSR and the log2 allelic fold change. The magnitude of the beta value has no direct biological interpretation as it is computed based on transformed expression values.* ***C)*** *Relationship between the regression beta values computed for each of the top eQTLs in the RPE and the log2 allelic fold change.* ***D)*** *Volcano plot of the log2 allelic fold change for each of the top eQTLs in NSR* ***E)*** *Volcano plot of the log2 allelic fold change for each of the top eQTLs in RPE*

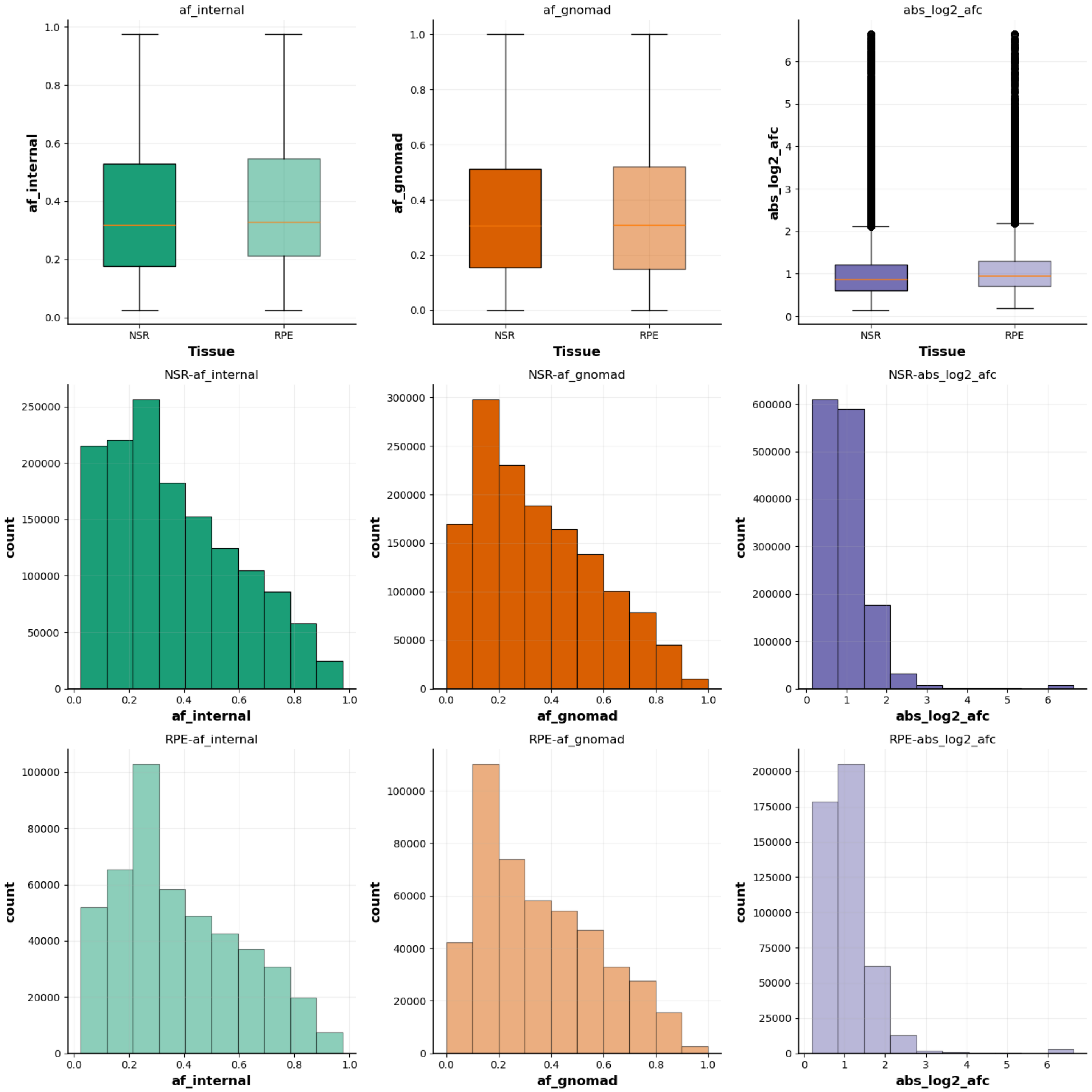

Supplementary Figure 7 Distribution of internal alternate allele frequencies (left), gnomAD v4.1 allele frequencies (middle) and the absolute effect size (log2 allelic fold change) (right) for all METR-eQTLs in NSR and RPE. *Box plots show the median (centre line), interquartile range (box; Q1–Q3), and whiskers extending to the most extreme data points within 1.5×IQR. Points beyond the whiskers are plotted as outliers.*

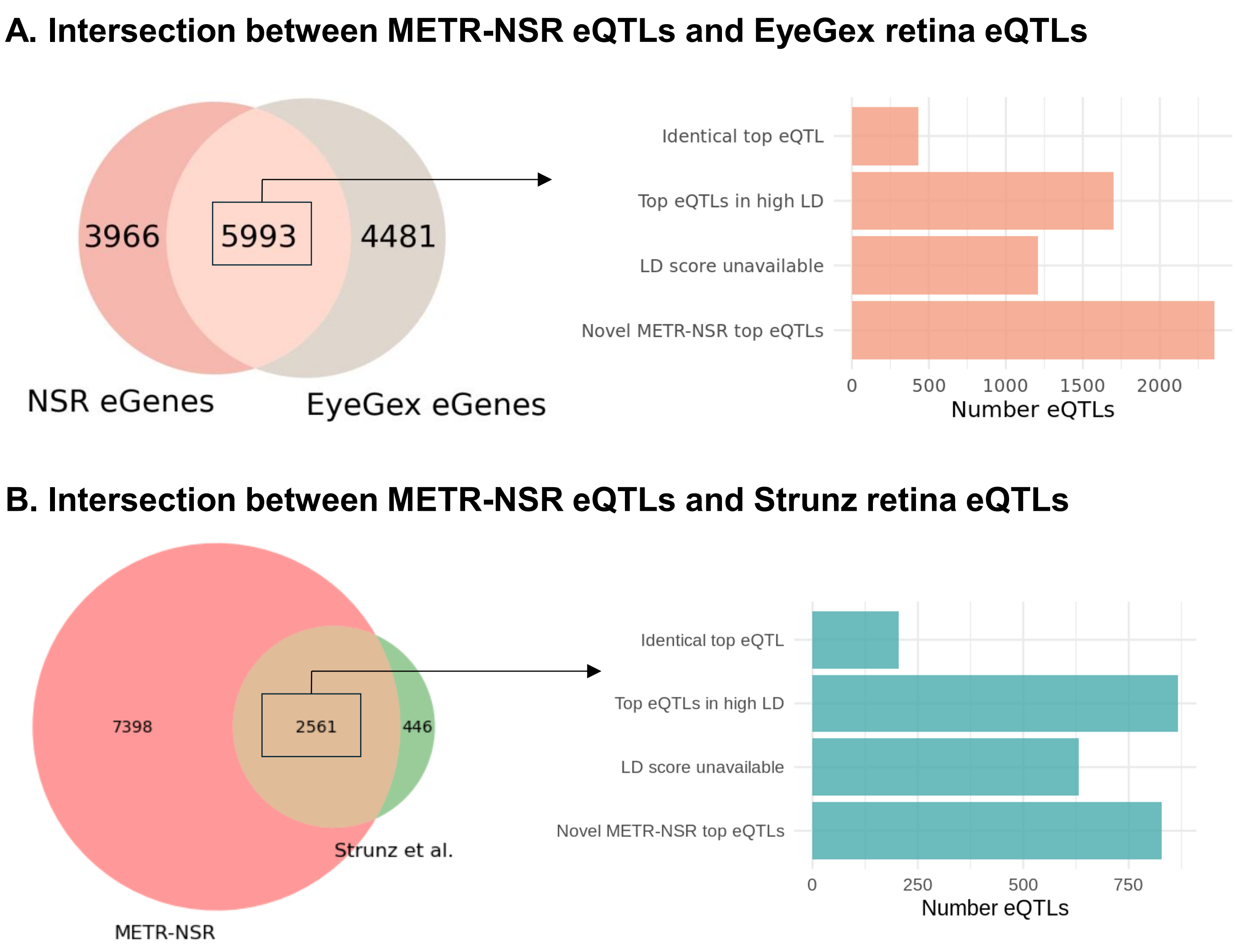

Supplementary Figure 8 Pairwise comparisons between METR-eGenes identified in the neurosensory retina (METR-NSR) and eGenes identified by EyeGex (A) and Strunz et al. (B). *In each panel we display the overlap of eGenes (left) and for eGenes that were present in both studies, we compared the respective top eQTLs to identify 1) eQTLs which were replicated in an additional study/studies; 2) eGenes where the top eQTL from each study was in high LD with each other (r2 > 0.8) and 3) eGenes where the top NSR hit was novel. For a subset of eQTLs that were tested for LD, the LD score was unavailable.*

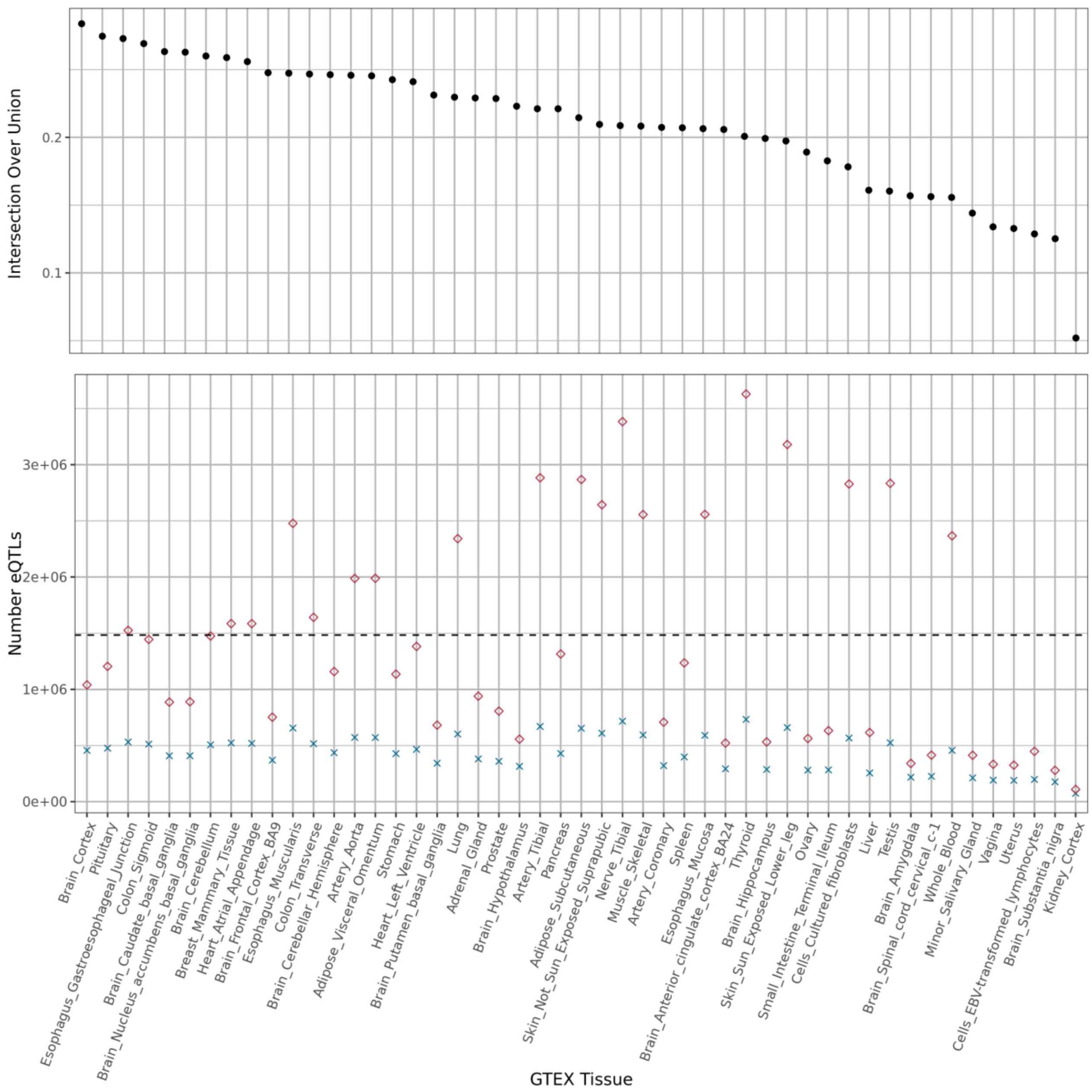

Supplementary Figure 9 *Intersection of the eQTLs identified in NSR (n = 183) and/or RPE (n = 176) with each GTEx study (v8). The bottom plot indicates the number of shared eQTLs in NSR/RPE and each tissue (cross) and the overall number of eQTLs identified in each GTEx study (circle). The top plot indicates relative proportion of eQTLs identified in each tissue and our study (intersection over union).*

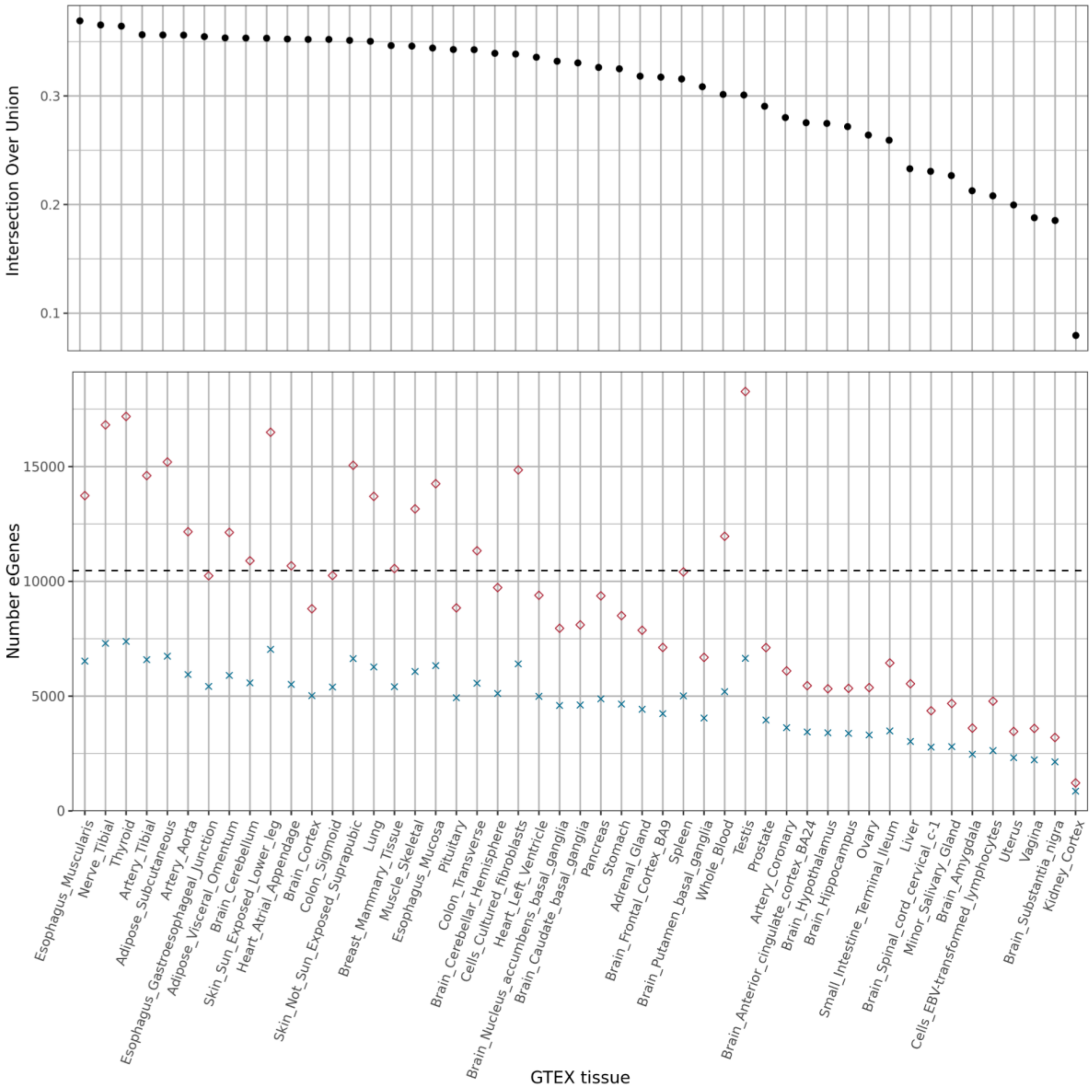

Supplementary Figure 10 Intersection between eGenes in each GTEX tissue and the METR-eGenes identified in the NSR (n = 183) and/or RPE (n = 176). *). The bottom plot indicates the number of shared eGenes in NSR/RPE and each tissue (cross) and the overall number of eGenes identified in each GTEx study (circle). The top plot indicates relative proportion of eGenes identified in each tissue and our study (intersection over union).*

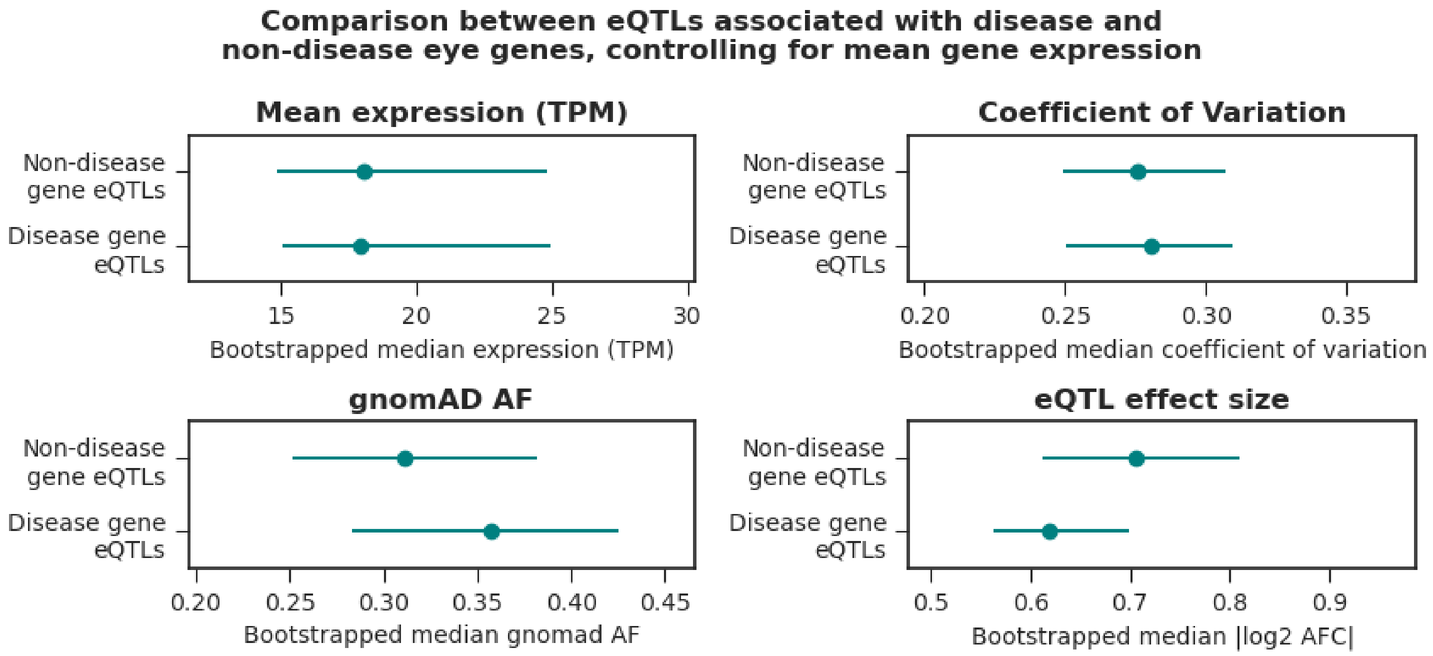

Supplementary Figure 11 Bootstrapping analysis indicated that eQTLs associated with known monogenic eye disease genes have higher allele frequencies and lower effect sizes, when controlling for mean expression. *Each panel shows the bootstrapped mean and 95% confidence intervals generated after 1000 iterations of subsampling. In each subsample we selected 100 random eQTLs associated with eye disease genes and another 100 which were associated with genes that are not known to cause monogenic eye disorders, matched for gene expression (+- 5% TPM).*

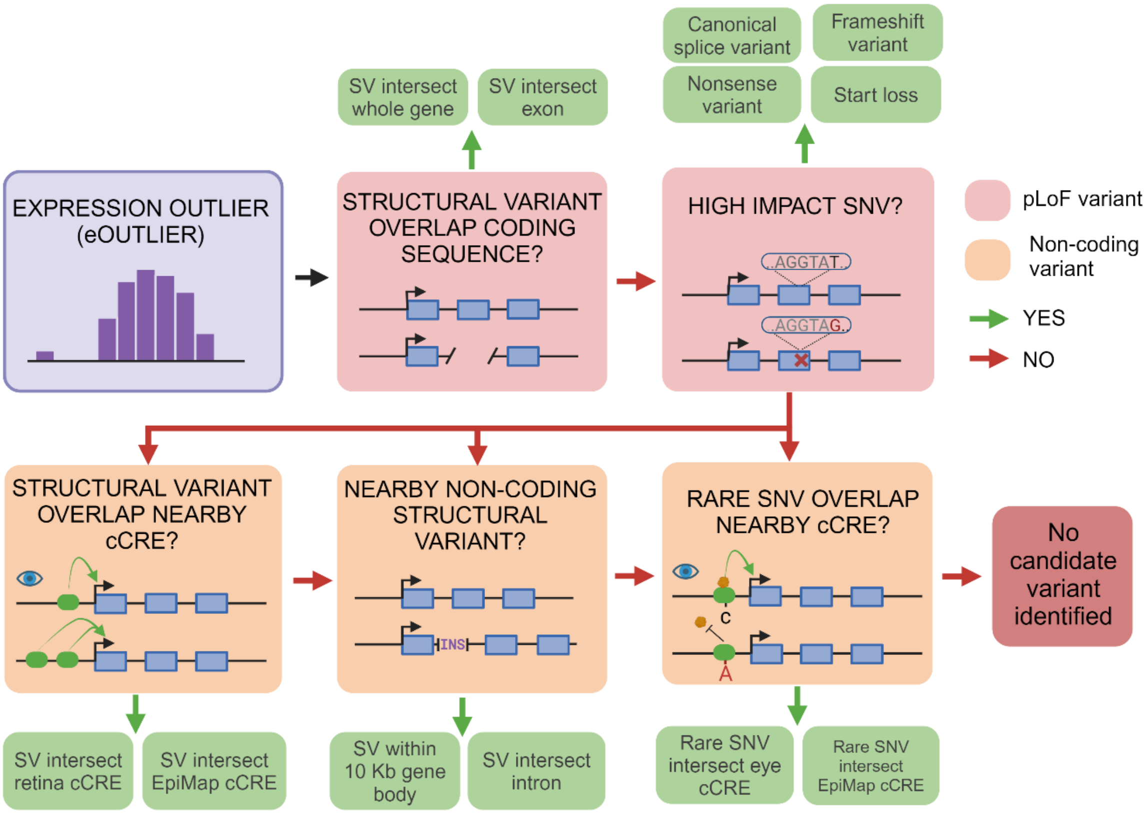

Supplementary Figure 12 Overview of the hierarchical workflow to identify candidate variants driving outlier expression *Created in BioRender. Ellingford, J. (2026) https://BioRender.com/fhy2fft*

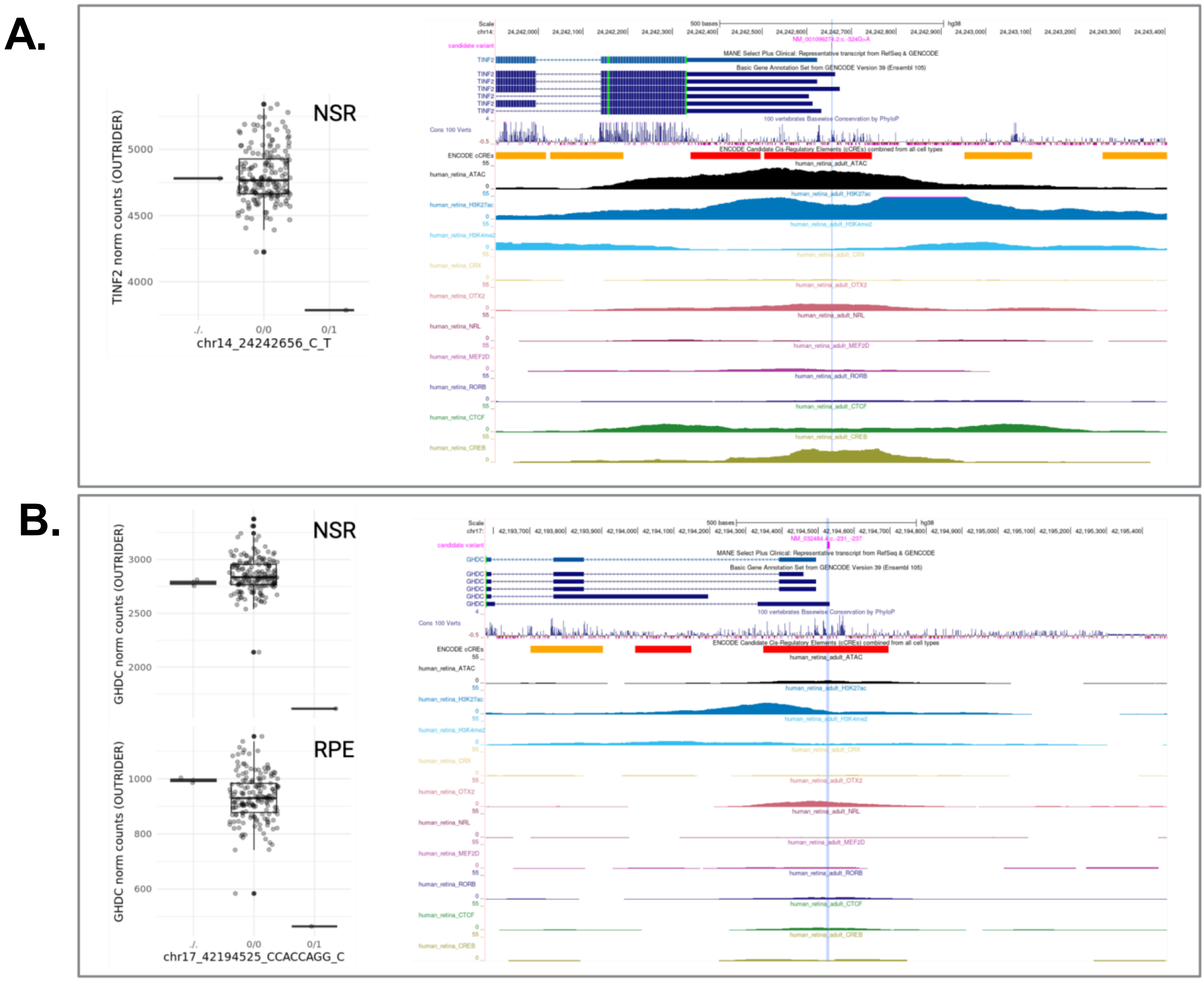

Supplementary Figure 13 Candidate rare small variants driving METR transcriptomic outliers in neurosensory retina (NSR) and retinal pigment epithelium (RPE). *In each caption, the tissue and relative outlier expression profiles calculated through OUTRIDER for individuals with missing (./.), homozygous reference (0/0) and heterozygous alternate (0/1) genotypes are shown, alongside genome tracks displaying transcript isoforms, evolutionary conservation and candidate cis-regulatory elements (cCREs) identified in EpiMap and Cherry et al. Box and whiskers plots show median values and interquartile ranges, with grey dots indicating normalised count values for single samples, and statistical outliers for each genotype indicated with black dots. In all examples, the variant intersects epigenomic peaks in retina and other tissues indicative of a promoter region for the MANE transcript:* ***A)*** *NM_001099274.2:c.-324G>A, TINF2;* ***B)*** *NM_032484.4:c.-231_237del, GHDC, outlier expression in both NSR and RPE*

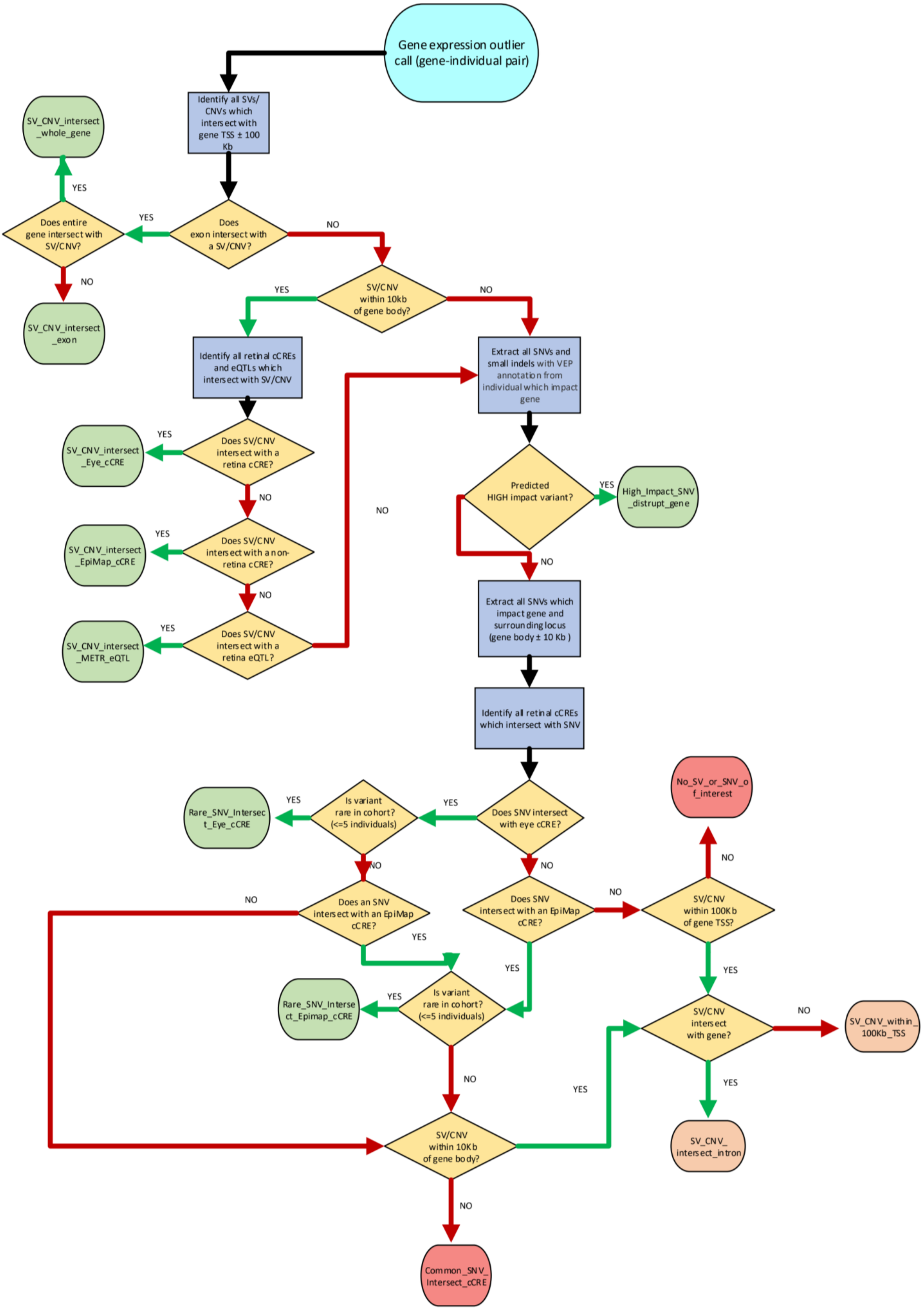

Supplementary Figure 14 Workflow used to identify candidate variants driving eOutlier events. *The workflow was automated using Snakemake, where each processing step was defined by an individual rule, and decision branches were defined using checkpoints.*

Supplementary References

1. Tap, J. *et al.* Effects of the long-term storage of human fecal microbiota samples collected in RNAlater. *Sci. Rep.* **9**, 601 (2019).

2. GTEx Consortium. Laboratory and Analysis Methods. (2019).

3. Dobin, A. *et al.* STAR: ultrafast universal RNA-seq aligner. *Bioinformatics* **29**, 15–21 (2013).

4. Picard toolkit. Broad Institute (2019).

5. Frankish, A. *et al.* GENCODE reference annotation for the human and mouse genomes. *Nucleic Acids Res.* **47**, D766–D773 (2019).

6. DeLuca, D. S. *et al.* RNA-SeQC: RNA-seq metrics for quality control and process optimization. *Bioinformatics* **28**, 1530–1532 (2012).

7. Li, B. & Dewey, C. N. RSEM: accurate transcript quantification from RNA-Seq data with or without a reference genome. *BMC Bioinformatics* **12**, 323 (2011).

8. Chu, T., Wang, Z., Pe'er, D. & Danko, C. G. Cell type and gene expression deconvolution with BayesPrism enables Bayesian integrative analysis across bulk and single-cell RNA sequencing in oncology. *Nat. Cancer* **3**, 505–517 (2022).

9. Monavarfeshani, A. *et al.* Transcriptomic analysis of the ocular posterior segment completes a cell atlas of the human eye. *Proc. Natl. Acad. Sci. U. S. A.* **120**, e2306153120 (2023).

10. Love, M. I., Huber, W. & Anders, S. Moderated estimation of fold change and dispersion for RNA-seq data with DESeq2. *Genome Biol.* **15**, 550 (2014).

11. Robinson, M. D., McCarthy, D. J. & Smyth, G. K. edgeR: a Bioconductor package for differential expression analysis of digital gene expression data. *Bioinformatics* **26**, 139–140 (2010).

12. Liao, Y., Wang, J., Jaehnig, E. J., Shi, Z. & Zhang, B. WebGestalt 2019: gene set analysis toolkit with revamped UIs and APIs. *Nucleic Acids Res.* **47**, W199–W205 (2019).

13. Sayols, S. rrvgo: a Bioconductor package for interpreting lists of Gene Ontology terms. *MicroPublication Biol.* https://doi.org/10.17912/micropub.biology.000811 (2023) doi:10.17912/micropub.biology.000811.

14. Krusche, P. *et al.* Best practices for benchmarking germline small-variant calls in human genomes. *Nat. Biotechnol.* **37**, 555–560 (2019).

15. Pedersen, B. S. *et al.* Somalier: rapid relatedness estimation for cancer and germline studies using efficient genome sketches. *Genome Med.* **12**, 62 (2020).

16. Robinson, M. D. & Oshlack, A. A scaling normalization method for differential expression analysis of RNA-seq data. *Genome Biol.* **11**, R25 (2010).

17. McCarthy, D. J., Chen, Y. & Smyth, G. K. Differential expression analysis of multifactor RNA-Seq experiments with respect to biological variation. *Nucleic Acids Res.* **40**, 4288–4297 (2012).

18. Stegle, O., Parts, L., Durbin, R. & Winn, J. A Bayesian Framework to Account for Complex Non-Genetic Factors in Gene Expression Levels Greatly Increases Power in eQTL Studies. *PLOS Comput. Biol.* **6**, e1000770 (2010).

19. Patterson, N., Price, A. L. & Reich, D. Population Structure and Eigenanalysis. *PLOS Genet.* **2**, e190 (2006).

20. Price, A. L. *et al.* Principal components analysis corrects for stratification in genome-wide association studies. *Nat. Genet.* **38**, 904–909 (2006).

21. Taylor-Weiner, A. *et al.* Scaling computational genomics to millions of individuals with GPUs. *Genome Biol.* **20**, 228 (2019).

22. Ongen, H., Buil, A., Brown, A. A., Dermitzakis, E. T. & Delaneau, O. Fast and efficient QTL mapper for thousands of molecular phenotypes. *Bioinformatics* **32**, 1479–1485 (2016).

23. Dudbridge, F. & Koeleman, B. P. C. Efficient Computation of Significance Levels for Multiple Associations in Large Studies of Correlated Data, Including Genomewide Association Studies. *Am. J. Hum. Genet.* **75**, 424–435 (2004).

24. Galwey, N. W. A new measure of the effective number of tests, a practical tool for comparing families of non-independent significance tests. *Genet. Epidemiol.* **33**, 559–568 (2009).

25. Storey, J. D. & Tibshirani, R. Statistical significance for genomewide studies. *Proc. Natl. Acad. Sci. U. S. A.* **100**, 9440–9445 (2003).

26. Mohammadi, P., Castel, S. E., Brown, A. A. & Lappalainen, T. Quantifying the regulatory effect size of cis-acting genetic variation using allelic fold change. *Genome Res.* **27**, 1872–1884 (2017).

27. Ratnapriya, R. *et al.* Retinal transcriptome and eQTL analyses identify genes associated with age-related macular degeneration. *Nat. Genet.* **51**, 606–610 (2019).

28. Strunz, T. *et al.* A mega-analysis of expression quantitative trait loci in retinal tissue. *PLoS Genet.* **16**, e1008934 (2020).

29. Phan, L. *et al.* The evolution of dbSNP: 25 years of impact in genomic research. *Nucleic Acids Res.* **53**, D925–D931 (2025).

30. Myers, T. A., Chanock, S. J. & Machiela, M. J. LDlinkR: An R Package for Rapidly Calculating Linkage Disequilibrium Statistics in Diverse Populations. *Front. Genet.* **11**, 157 (2020).

31. Orozco, L. D. *et al.* Integration of eQTL and a Single-Cell Atlas in the Human Eye Identifies Causal Genes for Age-Related Macular Degeneration. *Cell Rep.* **30**, 1246-1259.e6 (2020).

32. McLaren, W. *et al.* The Ensembl Variant Effect Predictor. *Genome Biol.* **17**, 122 (2016).

33. Cherry, T. J. *et al.* Mapping the cis-regulatory architecture of the human retina reveals noncoding genetic variation in disease. *Proc. Natl. Acad. Sci.* **117**, 9001–9012 (2020).

34. Wang, S. K. *et al.* Single-cell multiome of the human retina and deep learning nominate causal variants in complex eye diseases. *Cell Genomics* **2**, 100164 (2022).

35. Boix, C. A., James, B. T., Park, Y. P., Meuleman, W. & Kellis, M. Regulatory genomic circuitry of human disease loci by integrative epigenomics. *Nature* **590**, 300–307 (2021).

36. Snyder, M. P. *et al.* Perspectives on ENCODE. *Nature* **583**, 693–698 (2020).

37. Quinlan, A. R. & Hall, I. M. BEDTools: a flexible suite of utilities for comparing genomic features. *Bioinformatics* **26**, 841–842 (2010).

38. Lenassi, E. *et al.* EyeG2P: an automated variant filtering approach improves efficiency of diagnostic genomic testing for inherited ophthalmic disorders. *J. Med. Genet.* **60**, 810–818 (2023).

39. Schubach, M., Maass, T., Nazaretyan, L., Röner, S. & Kircher, M. CADD v1.7: using protein language models, regulatory CNNs and other nucleotide-level scores to improve genome-wide variant predictions. *Nucleic Acids Res.* **52**, D1143–D1154 (2024).
