# Supplementary material for: Paired DNA and RNA sequencing uncovers common and rare variation regulating human retinal gene expression
Source: Nat Commun. 2026 May 26;17:4595. doi: 10.1038/s41467-026-72979-4 (PMC13213049; doi:10.1038/s41467-026-72979-4)
Supplement: Supplementary file 2 — Description of Additional Supplementary Files [file 41467_2026_72979_MOESM2_ESM.pdf]

Description of Additional Supplementary Files

Supplementary Data 1: Variant-level quality control steps taken to process the aggregate VCF. The multisample aggregate VCF (n=201) generated by DRAGEN 4.0.3 Population Mode was post-processed to retain high quality variant calls only. The number of variants remaining after each filtering step (minor allele count > 1) is indicated to the right.

Supplementary Data 2: Number of copy number variants (CNVs) and structural variants (SVs) per sample in the METR cohort. Structural variants were subcategorised into deletions, insertions, duplications and other events captured by chromosomal breakends. Copy number variants were classified into LOSS or GAIN. Only variants which passed the DRAGEN SV / CNV filter (FILTER=PASS) were counted per sample.

Supplementary Data 3: Quality control metrics for whole genome sequencing (WGS) data and bulk RNAseq data for Neurosensory Retina and Retinal Pigment Epithelium for the METR cohort. WGS data was available for all 201 samples, and NSR and RPE data was available for 183 and 176 respectively.

Supplementary Data 4: Gene set enrichment analysis of differentially expressed genes in the retinal pigment epithelium (RPE). We performed gene set enrichment analysis of Gene Ontology(GO) biological process terms associated with the subset of differentially expressed genes upregulated in the RPE (FDR < 0.05) (n = 7,353) relative to all genes expressed in the RPE (mean TPM > 1). The table indicates all GO biological process terms that were enriched for this subset of genes (FDR < 0.05 and enrichment ratio > 1) . GO terms were subsequently clustered based on semantic similarity. For each GO term, we indicate its cluster ID and the corresponding cluster parent term.

Supplementary Data 5: Gene set enrichment analysis of differentially expressed genes in the neurosensory retina (NSR). We performed gene set enrichment analysis of gene ontology (GO) biological process terms associated with the subset of differentially expressed genes upregulated in the NSR (FDR < 0.05) (n = 7,604) relative to all genes expressed in the NSR (mean TPM > 1). The table indicates all GO biological process terms that were enriched for this subset of genes (FDR < 0.05 and enrichment ratio > 1) . GO terms were subsequently clustered based on semantic similarity. For each GO term, we indicate its cluster ID and the corresponding cluster parent term.

Supplementary Data 6: Candidate variants driving eOutlier events in the NSR and the RPE identified by a hierarchical model (decision tree) and/or a probabilistic model (Watershed). Each eOutlier event captured by OUTRIDER is described by the fold change in gene expression and the corresponding false-discovery rate adjusted p-value. OUTRIDER was run independently for the NSR and RPE, so eOutliers can be tissue-specific or common to both tissues. The prioritised variants identified by the hierarchical model (decision tree) include small variants (SNVs and indels), structural variants and copy number variants. Watershed was only run on small variants. Each variant was assigned a category, and some

variants (CNVs, SVs and pLoF SNVs) were assigned a subcategory. If a variant was not identified by one of the methods, then the category 'no_candidate_variant' is indicated.

Supplementary Data 7: Annotations used by the Watershed model. Annotations were sourced from Ensembl's Variant Effect Predictor, CADD, EpiMap and curated retina cCREs from Cherry et al. If an annotation was missing for a particular variant, then a default value (recommended by CADD) was used instead
