# Supplementary material for: Paired DNA and RNA sequencing uncovers common and rare variation regulating human retinal gene expression
Source: Nat Commun. 2026 May 26;17:4595. doi: 10.1038/s41467-026-72979-4 (PMC13213049; doi:10.1038/s41467-026-72979-4)
Supplement: Supplementary file 4 — Reporting Summary [file 41467_2026_72979_MOESM4_ESM.pdf]

nature portfolio

Corresponding author(s): Jamie Ellingford

Last updated by author(s): 18/11/2025

Reporting Summary

Nature Portfolio wishes to improve the reproducibility of the work that we publish. This form provides structure for consistency and transparency in reporting. For further information on Nature Portfolio policies, see our Editorial Policies and the Editorial Policy Checklist.

Statistics

For all statistical analyses, confirm that the following items are present in the figure legend, table legend, main text, or Methods section.

n/a	Confirmed	
☐	☒	The exact sample size (*n*) for each experimental group/condition, given as a discrete number and unit of measurement
☐	☒	A statement on whether measurements were taken from distinct samples or whether the same sample was measured repeatedly
☐	☒	The statistical test(s) used AND whether they are one- or two-sided<br>*Only common tests should be described solely by name; describe more complex techniques in the Methods section.*
☐	☒	A description of all covariates tested
☐	☒	A description of any assumptions or corrections, such as tests of normality and adjustment for multiple comparisons
☐	☒	A full description of the statistical parameters including central tendency (e.g. means) or other basic estimates (e.g. regression coefficient) AND variation (e.g. standard deviation) or associated estimates of uncertainty (e.g. confidence intervals)
☐	☒	For null hypothesis testing, the test statistic (e.g. *F*, *t*, *r*) with confidence intervals, effect sizes, degrees of freedom and *P* value noted<br>*Give P values as exact values whenever suitable.*
☐	☒	For Bayesian analysis, information on the choice of priors and Markov chain Monte Carlo settings
☒	☐	For hierarchical and complex designs, identification of the appropriate level for tests and full reporting of outcomes
☐	☒	Estimates of effect sizes (e.g. Cohen's *d*, Pearson's *r*), indicating how they were calculated

Our web collection on statistics for biologists contains articles on many of the points above.

Software and code

Policy information about availability of computer code

Data collection	RNA sequencing data was processed following the GTEX v.8 pipeline, which included the following tools: Picard v.2.27.1, RNA-SeQC v.1.19, RSEM v.1.3.0, STAR v.2.7.4. Whole-Genome Sequencing data was processed with Illumina DRAGEN v.3.7.8 with Population Mode enabled.
Data analysis	We used the following command-line interface tools for data analysis: bedtools v.2.3.1, bcftools v.1.16, conda v.24.11.3, DROP v.1.4.0, EIGENSOFT v.6.0.1, jupyterlab v. 3.6.6, PLINK v2.0, samtools v.1.21, snakemake v.7.32, somalier v.0.2.18, tensorQTL v. 1.0.7 and vim v. 9.1. Python v.3.12.10 was used with the following packages: numpy v.2.3.3, scipy v.1.16.2, pandas v.2.3.2, matplotlib v.3.10.6, seaborn v. 0.13.2, scikit-learn v.1.7.2, pybedtools v.0.12.0 and pygenometracks v.3.3. R v.4.5.0 and RStudio v.2025.05.0 were used with the following packages: deseq2, edgeR, ggplot2, tidyverse, BayesPrism, Watershed, WebGestalt. All required dependencies to run the above packages were also installed.

For manuscripts utilizing custom algorithms or software that are central to the research but not yet described in published literature, software must be made available to editors and reviewers. We strongly encourage code deposition in a community repository (e.g. GitHub). See the Nature Portfolio guidelines for submitting code & software for further information.

Data

Policy information about availability of data

All manuscripts must include a data availability statement. This statement should provide the following information, where applicable:

- Accession codes, unique identifiers, or web links for publicly available datasets
- A description of any restrictions on data availability
- For clinical datasets or third party data, please ensure that the statement adheres to our policy

All raw RNA sequencing and genomic sequencing datasets generated in this study have been made available through the European-Genome phenome Archive (EGA; Study ID: EGAS50000001443; Dataset: EGAD50000002082). Processed datasets, including eQTL results, eOutlier statistics and aggregated genomic variant files are also available through EGA.

Research involving human participants, their data, or biological material

Policy information about studies with human participants or human data. See also policy information about sex, gender (identity/presentation), and sexual orientation and race, ethnicity and racism.

Reporting on sex and gender	While sex was an important consideration in the study design, the post-mortem eye samples that were included in our cohort were selected based on RNA integrity quality-control metrics. The QC process was performed without knowledge of sample sex.<br>In the manuscript we describe the genetically inferred sex karyotypes of the donors in our cohort. The eQTL mapping and eOutlier analysis was performed on all samples in the cohort so findings apply to both sexes.<br>Due to power limitations, we were unable to obtain meaningful results by running these analyses on a disaggregated cohort by sex. However, we included sex as a covariate in the eQTL model to control for differences in gene expression and regulation driven by biological sex.<br>Additionally, we have included sex metadata in the sequencing datasets that have been made available through the European-Genome phenome Archive (EGA).
Reporting on race, ethnicity, or other socially relevant groupings	Self-reported data on race and ethnicity were unavailable for the post-mortem eye samples that were used in this study. The included eye samples were selected based on RNA integrity quality-control metrics without knowledge of sample race/ ethnicity.<br>In the manuscript, we described the inferred genetic-ancestry of all donors in our cohort based on principal component analysis (PCA) of genotype data of our cohort compared to PCA analysis of the 1000 Genomes cohort. We used the same super-population groups that were included in the 1000 Genomes Project to describe inferred genetic ancestry
Population characteristics	The median age of our cohort was 71 (IQR 64-77), with a slight male slight male predominance (63.7%). The median ischemic time was 40 hours (IQR=32-44). While 47 individuals (23% of the cohort) were found to carry genetic variants that confer high-risk for age-related macular degeneration (AMD), none of the 201 individuals included in the cohort had phenotypic presentation, assessed post-mortem, consistent with late-stage AMD or monogenic ophthalmic disorders.
Recruitment	The eye samples utilised in this study were obtained from the Manchester Eye Tissue Repository, a non-profit tissue bank. No compensation was provided for receipt or delivery of tissue samples. Eye samples that were included in our cohort were selected exclusively based on RNA integrity quality-control metrics.
Ethics oversight	All research and approaches undertaken in this manuscript were approved by the North West – Greater Manchester Central Research Ethics Committee and NHS Health Research Authority (15/NW/0932).

Note that full information on the approval of the study protocol must also be provided in the manuscript.

Field-specific reporting

Please select the one below that is the best fit for your research. If you are not sure, read the appropriate sections before making your selection.

☒ Life sciences ☐ Behavioural & social sciences ☐ Ecological, evolutionary & environmental sciences

For a reference copy of the document with all sections, see nature.com/documents/nr-reporting-summary-flat.pdf

Life sciences study design

All studies must disclose on these points even when the disclosure is negative.

Sample size	201
Data exclusions	Relevant phenotypic, accessibility and quality control metrics that are further described in the manuscript. Eye samples were selected on the basis of high RNA-integrity scores and absence of phenotypic indications of monogenic opthalmic disorders and late-stage AMD. We excluded samples with outlier WGS QC metrics, including median coverage, number of reads with Q>30, percentage genome >15x coverage, uniformity of coverage, total number variants, total number of SNVs, transition/transversion ratio and heterozygous/homozygous ratio. We excluded RNAseq samples with outlier quality RNAseq QC metrics, including total number of reads, read length, 3'/5' bias.

Replication	Assessment against previously published eQTL studies; within cohort replication of eOutlier genes and variant prioritisation; concordance of bespoke cellular assays with associations determined from donated samples
Randomization	Some randomizaion was performed to enable appropriate confidence to be drawn from observations regarding properties of eVariants associated with known disease genes and non-disease genes. This randomization was informed by overall gene expression level and gene characteristics, such as density of transcription start sites within a given genomic interval
Blinding	no blinding was performed for this study.

Reporting for specific materials, systems and methods

We require information from authors about some types of materials, experimental systems and methods used in many studies. Here, indicate whether each material, system or method listed is relevant to your study. If you are not sure if a list item applies to your research, read the appropriate section before selecting a response.

Materials & experimental systems

n/a	Involved in the study	
☒	☐	Antibodies
☐	☒	Eukaryotic cell lines
☒	☐	Palaeontology and archaeology
☒	☐	Animals and other organisms
☒	☐	Clinical data
☒	☐	Dual use research of concern
☒	☐	Plants

Methods

n/a	Involved in the study	
☒	☐	ChIP-seq
☒	☐	Flow cytometry
☒	☐	MRI-based neuroimaging

Eukaryotic cell lines

Policy information about cell lines and Sex and Gender in Research

Cell line source(s)	*Human K562 cell line*
Authentication	*The cell line was not authenticated*
Mycoplasma contamination	*The cell line was not tested for mycoplasma contamination*
Commonly misidentified lines (See ICLAC register)	*As far as the authors are aware there are no commonly misidentified cell-lines associated with human K562 cells.*

Plants

Seed stocks	*N/A*
Novel plant genotypes	*N/A*
Authentication	*N/A*
